# In Silico Approach to Design of New Multi-Targeted Inhibitors Based on Quinoline Ring with Potential Anticancer Properties

**DOI:** 10.3390/ijms26104620

**Published:** 2025-05-12

**Authors:** Żaneta Czyżnikowska, Martyna Mysłek, Aleksandra Marciniak, Remigiusz Płaczek, Aleksandra Kotynia, Edward Krzyżak

**Affiliations:** 1 Department of Basic Chemical Sciences, Faculty of Pharmacy, Wroclaw Medical University, Borowska 211, 50-556 Wroclaw, Poland; aleksandra.marciniak@umw.edu.pl (A.M.); remigiusz.placzek@student.umw.edu.pl (R.P.); aleksandra.kotynia@umw.edu.pl (A.K.); edward.krzyzak@umw.edu.pl (E.K.); 2 “Biomolecule” Student Science Club, Department of Basic Chemical Sciences, Faculty of Pharmacy, Wroclaw Medical University, Borowska 211, 50-556 Wroclaw, Poland

**Keywords:** anticancer drugs, multi-target inhibitors, design of new pharmaceuticals, CADD, molecular docking

## Abstract

Searching for new anticancer drugs is a significant challenge for the medical community due to the current limitations of existing treatments. The primary objective of this study was to design and optimize multi-targeted drug candidates based on a quinoline scaffold. In this paper, we adopt various in silico techniques, including molecular docking, molecular dynamics simulations, and ADMET property modeling, to predict the binding affinity and interactions of 7-ethyl-10-hydroxycamptothecin derivatives with multiple biological targets. The interactions of these compounds with three potential molecular targets, topoisomerase I, bromodomain-containing protein 4, and ATP-binding cassette sub-family G member 2 proteins, were analyzed. It has been previously proved that the inhibition of these molecular targets may have beneficial effects on cancer treatment. The designed chemical compounds can effectively interact with selected proteins, thereby establishing their potential as drug candidates. Molecular docking revealed promising binding affinities, with topoisomerase I docking scores ranging from −9.0 to −10.3 kcal/mol, BRD4 scores from −6.6 to −8.0 kcal/mol, and ABCG2 scores from −8.0 to −10.0 kcal/mol. Furthermore, the ADMET property analysis indicates promising pharmacological profiles, protein binding affinity, selectivity, and bioavailability while minimizing toxicity. For example, satisfactory logP values have been demonstrated in the favorable range for bioavailability after oral administration. Additionally, several compounds exhibited predicted aqueous solubility values greater than −3, suggesting moderate-to-good solubility, which is crucial for oral drug delivery.

## 1. Introduction

It is well known that cancer is one of the leading causes of death worldwide. The increasing aggressiveness of this group of diseases, their biological complexity, and growing drug resistance makes searching for new anti-cancer agents and new molecular targets a major task for modern medicine. The development of multi-target anticancer drugs has become a crucial strategy in oncology to overcome the limitations of single-targeted therapies. Multi-target agents are designed to act simultaneously on multiple critical pathways or targets within cancer cells. Additionally, such therapy lowers the risk of adverse drug interactions. In the present study, we selected three important molecular targets for anticancer drugs. A promising strategy is the synergistic inhibition of TOPO-I, BRD4, and ABCG2, which involves the targeting of three critical mechanisms in cancer progression. Topoisomerase I plays a vital role in DNA replication, and its inhibition disrupts cancer cell proliferation. However, cancer cells often develop resistance by enhancing DNA repair mechanisms. Bromodomain-containing protein 4 is a key regulator of oncogene expression and cancer progression, and its inhibition has been shown to impair cancer cell proliferation, disrupt the cell cycle, and induce apoptosis. ATP-binding cassette sub-family G member 2 contributes to multidrug resistance by effluxing anticancer drugs from cells. Inhibiting ABCG2 has been shown to enhance the efficacy of TOPO-1 inhibitors, addressing the challenge of drug resistance. Therefore, the simultaneous targeting of these three proteins could provide a synergistic approach to enhance cancer therapy and improve treatment outcomes (protein structure and function were presented in the Supplementary; see Figures S1–S3).

As a reference compound, we used 7-ethyl-10-hydroxycamptothecin (SN-38). It is a pharmaceutical agent that has been demonstrated to possess anticancer properties. SN-38 has proven activity as an inhibitor of all three proteins mentioned, but it is the weakest relative to ABCG2 [1,2,3]. It represents an active metabolite of irinotecan, a prodrug that is frequently employed in the treatment of cancer [3]. Irinotecan is a topoisomerase I inhibitor that received approval for use in patients in 1994. It has been demonstrated to exhibit a broad spectrum of anticancer activity in a range of cancers, including lung, colorectal, cervical, and ovarian. The metabolite SN-38, which was selected as the initial structural template, has been shown to exhibit a potency that is 100–1000 times greater than that of the prodrug [3,4]. There is considerable optimism among the scientific community about the design and utilization of analogs of the SN-38 compound [3,5]. The structural motifs of SN-38 are responsible for its biological activity (See Section 2.1). Therefore, considering the pharmacophore properties of the reference compound, we have designed a series of 12 new 2,3-dimethoxy-*N*-(4-methylquinolin-3-yl)cyclopentanecarboxamide derivatives.

The computational techniques of drug design offer the development of promising and effective compounds for the treatment of complex diseases that involve the dysregulation of multiple molecular pathways. Molecular modeling and virtual screening strategies are employed to explore the broad therapeutic potential of a large group of compounds, taking into account new derivatives and existing therapeutics by identifying key protein targets and their interconnections within disease-relevant signaling networks. It is estimated that using the traditional approach can extend this process to 17 years and can generate costs of nearly USD 3 trillion. In addition, the success rate is around 10%. Two main approaches have been used in computer-aided drug design. One is Structure-Based Drug Design (SBDD), and the other is Ligand-Based Drug Design (LBDD) [6]. Since quantitative structure–activity relationship models (QSAR) are known to be excellent tools to support the drug design process, several internet-based programs have emerged that use high-quality experiments and allow for the performance of multiple drug-likeness analyses and the predictions of many ADMET properties [7].

Numerous studies have demonstrated the successful application of these methodologies in identifying anticancer agents that target key proteins involved in tumour progression, such as BRD4 and ABCG2 [8,9]. These examples highlight the effectiveness of purely computational protocols in the early stages of drug development, especially when the aim is to identify multi-target compounds for complex diseases such as cancer.

The objective of our study was to design new compounds based on the quinoline scaffold that would demonstrate potential anticancer properties and could interact with more than one molecular target. In this regard, we used several theoretical approximations that allowed us to propose promising candidates and analyze their anticancer potential. All designed compounds were docked to three molecular targets, and their ADMET properties were analyzed. The results identify all compounds as promising candidates based on their binding potency and drug-likelihood parameters. Consequently, molecular dynamics simulations were performed to analyze dynamic aspects of interactions between compounds and selected molecular targets.

## 2. Results and Discussion

### 2.1. Designing Strategy

#### 2.1.1. Molecular Targets

**Figure 1:**
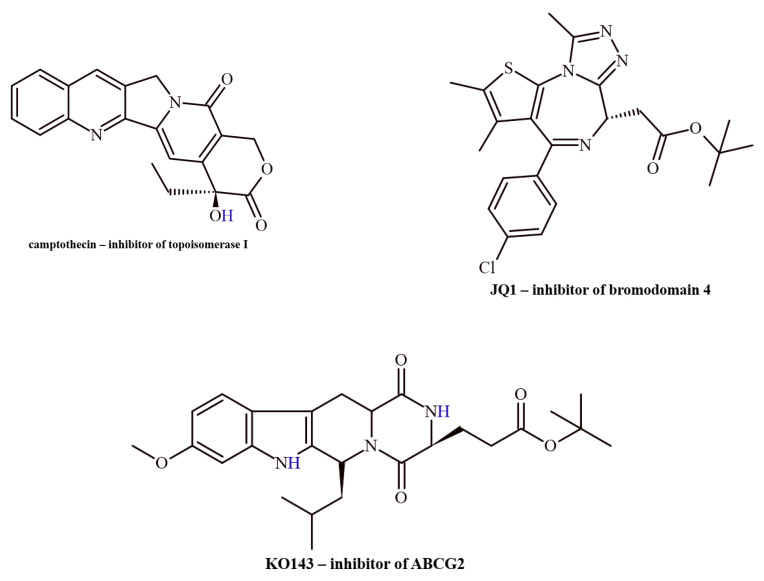
Examples of inhibitors for selected molecular targets.

Three proteins that play a role in carcinogenesis and/or metastasis were selected as molecular targets: topoisomerase I, bromodomain-containing protein 4 (BRD4), and ATP-binding cassette sub-family G member 2 protein (ABCG2). Selected inhibitors for chosen molecular targets are presented in Figure 1.

To achieve high specificity and affinity to the active sites of the molecular targets, the structure of the inhibitors should have the appropriate chemical and geometric features and possess suitable allosteric properties. The binding site of topoisomerase I has a structural conformation that enables it to recognize and interact with the major groove of DNA. This interaction occurs mainly via the positively charged amino acid residues and negatively charged phosphate groups of DNA [10]. The special “clamp-like” conformation of the protein opens and closes around the DNA, which allows for control of the strands. The structural flexibility of the binding cavity allows it to adapt to different DNA conformations. Designed inhibitors can exploit these structural and functional aspects to block topoisomerase activity, often by stabilizing the enzyme–DNA complex or preventing the enzyme from completing its catalytic cycle. For example, camptothecin and its derivatives stabilize the covalent enzyme–DNA intermediate and prevent DNA religation, which leads to DNA breaks and ultimately inhibits replication and transcription [11]. Many inhibitors bind to the hydrophobic pocket planar due to the aromatic rings that can stack with DNA bases within the cleavage complex, stabilizing the interaction. Some inhibitors can act as intercalators, which can distort the DNA, enhancing the enzyme’s binding but preventing it from dissociating normally. Another mechanism of action is the formation of hydrogen bonds and electrostatic interactions with specific amino acids within the topoisomerase active site [12]. Finally, the flexibility of topoisomerase I enables drugs to bind at different conformational states or non-catalytic allosteric sites, modulating enzyme activity.

The binding site of BRD4 contains two conserved bromodomains (BD1 and BD2), each with a binding pocket specific for acetylated lysine residues on histone tails. The hydrophobic acetyl-lysine binding site contains conserved amino acid residues, including asparagine, which participates in van der Waals interactions and forms hydrogen bonds with the acetylated amide group of lysine. Quite important stabilizing factors are electrostatic interactions inside the pocket with the histone tail. Similar to topoisomerase I, the BRD4 binding cavity is characterized by a certain degree of flexibility, which allows the protein to adapt to different histone acetylation patterns [13]. BRD4 inhibitors are small, branched molecules that mimic the acetyl-lysine structure, fitting into the binding pocket and competitively blocking BRD4 function. They bind to the hydrophobic cavity and often form a crucial hydrogen bond with a conserved asparagine residue, which is also involved in binding natural acetyl-lysine on histones. The position of inhibitors is stabilized through additional van der Waals interactions (for example, JQ1, OTX015) [14].

ATP-binding cassette sub-family G member 2 protein has a drug binding pocket localized in the transmembrane domain, where the hydrophobic environment allows for the accommodation of lipophilic compounds. Due to the character of amino acid residues in the membrane-spanning domains, non-polar interactions with hydrophobic inhibitors are possible. The key amino acid residues responsible for the stabilization of the complex are phenylalanine, tryptophan, and tyrosine, which participate in π–π stacking interactions. In addition, aliphatic residues such as leucine and valine contribute to the hydrophobic interactions that privilege the binding of lipophilic compounds. Polar amino acids can interact with charged anionic-type inhibitors [15]. The binding pocket of ABCG2 demonstrates affinity to a wide range of sizes and shapes of substrates, which contributes to its role in multidrug resistance. Fumitremorgin C is a potent inhibitor of protein that can bind to the transmembrane region by hydrophobic and hydrogen bonding interactions with key residues [16].

#### 2.1.2. Reference Compounds

**Figure 2:**
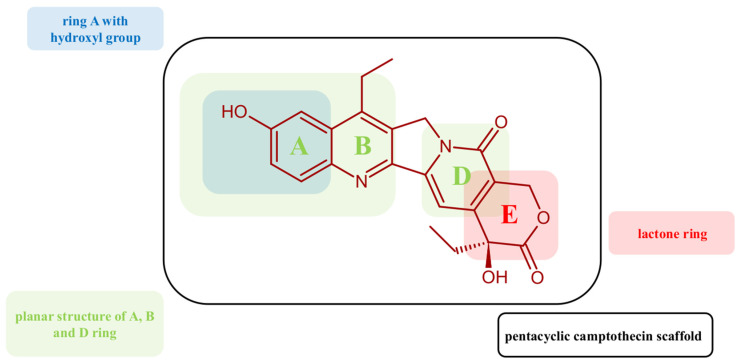
Structural features of SN-38 (**M0**) are responsible for binding to the molecular target.

As we mentioned in the introduction, as a reference compound, we used, in this study, 7-ethyl-10-hydroxycamptothecin (SN-38, marked in the work as **M0**). SN-38 has several characteristic structural motifs responsible for its biological activity. One of these is primarily the pentacyclic camptothecin scaffold, which provides the rigidity and flat structure needed for intercalation between DNA base pairs and positioning inhibitors to interact with the DNA–topoisomerase complex effectively. The lactone ring, with hydrophobic interactions and hydrogen bonding within the topoisomerase I active site, stabilizes the cleavage complex, preventing DNA religation (Figure 2, Ring E). The hydroxyl group on the B ring enhances its potency compared to irinotecan. This structural motif can form hydrogen bonds with amino acid residues in the topoisomerase I active site, stabilizing the cleavage complex. The planar structure of A, B, and D rings allows for effective DNA intercalation, blocking replication fork progression. One more important structural feature is the absence of large substituents. SN-38 lacks bulky groups, allowing it to readily interact with the DNA–topoisomerase I complex without the need for activation (see Figure 2). In vitro studies have shown that SN-38 can bind to BRD4 and be an inhibitor. The determined IC_50_ was IC_50_ = 547.7 nM. The inhibition process is reversible. SN-38 can stabilize BRD4 in cells and can significantly suppress the proliferation of the human leukemic cell K562. Furthermore, SN-38 can induce apoptosis in a dose-dependent manner and inhibit the expression of the BRD4 substrate C-MYC, as well as induce caspase-3 cleavage and BAX expression. It has been shown that carbonyl and hydroxyl groups play an important role in SN-38 activity by forming hydrogen bonds. SN-38 is a relatively weak inhibitor of ABCG2. It does not induce significant conformational changes in the protein, which determines its limited inhibitory potency. Its activity is similar to that of mitoxantrone but much weaker than that of imatinib. Both drugs bind to the transported substrate binding site and block the action of ABCG2. When ABCG2 is in an inward-facing conformation, imatinib, mitoxantrone, and SN-38 bind in the gap between the ABCG2 monomers, where they are sandwiched between the side chains of Phe439. While imatinib acts as a wedge to prevent ATP-induced NBD dimerization and TM helix collapse, the substrates mitoxantrone and SN-38 can move out of their initial binding sites and be translocated.

#### 2.1.3. Designed Compounds

**Figure 3:**
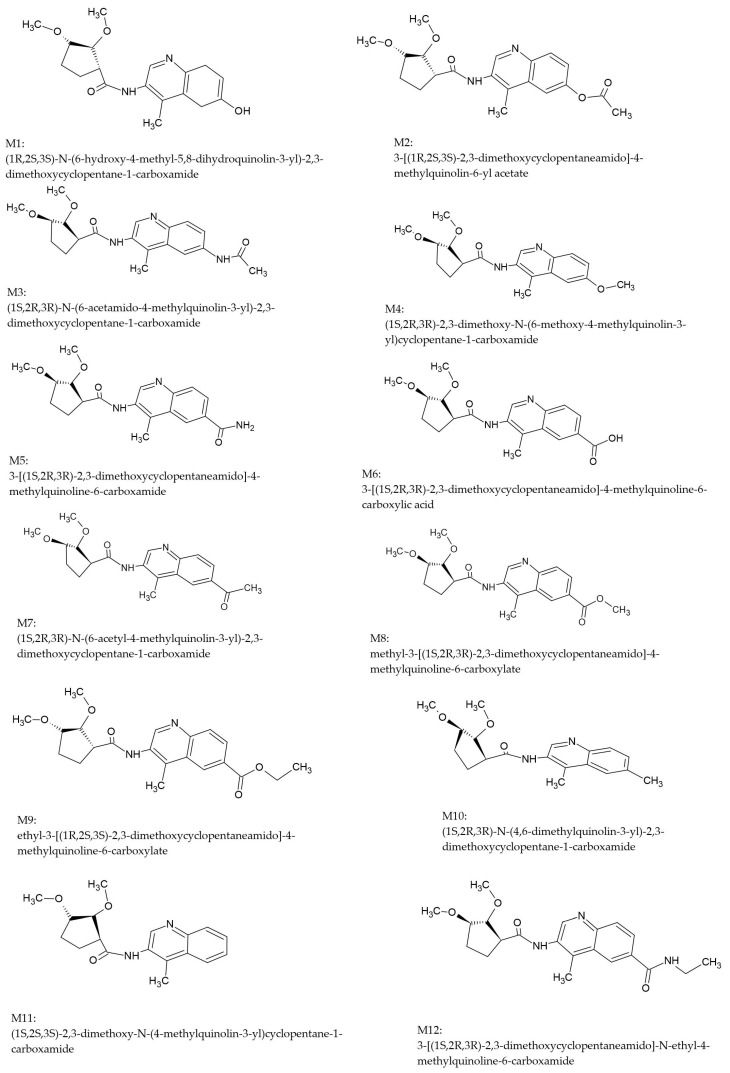
Structures of the designed compounds **M1**–**M12**.

Considering the structure of the binding sites of selected molecular targets and the pharmacophore properties of their inhibitors, we have designed a series of 12 new derivatives of 2,3-dimethoxy-*N*-(4-methylquinolin-3-yl)cyclopentanecarboxamide (see Figure 3). The common structural scaffold of the proposed inhibitors was a heterocyclic quinoline ring with a methyl substituent, which corresponds to the A and B rings of SM-38. A further common feature of all ligands was the presence of an amide bond linking a quinoline ring and a 2,3-dimethoxycyclopentane group. Its enforced spatial conformation mimics the central fragment of SN-38 and allows hydrogen bonding at the molecular target binding sites. Finally, all compounds possess a 2,3-dimethoxycyclopentane moiety with similar pharmacophore features as rings D and E of SN-38. The series was generated by modifying the substituents at position 7 of the quinoline ring. The novel derivatives presented in Figure 3 possess three chiral centers in the 2,3-dimethoxycyclopentane moiety. These are of crucial importance since they influence the spatial orientation of the pharmacophores and the overall stereochemical interaction between the ligands and the chosen molecular targets. As is well known, chirality plays a critical role in the process of drug design due to the different biological activities of molecules of different enantiomers. To ensure targeted therapeutic action and minimize adverse effects, it is important to employ stereoselective synthesis and chiral resolution techniques in the development of enantiomerically pure compounds.

### 2.2. Binding Properties of Designed Compounds Based on Molecular Docking

To analyze the binding mode of designed compounds and free energy of binding, we perform molecular docking using Autodock 4.2 [17]. The proposed inhibitors were docked, respectively, to topoisomerase I, BRD4, and ABCG2. The scoring functions are used to evaluate, approximately, the binding affinity between two molecules. As we know, they are particularly well-suited to the rank ordering of compounds according to their potencies. The scoring function, including the value of ΔG binding, which characterizes the affinity of TOPO-I/designed ligand complexes, varies from −9.0 to −10.0 kcal/mol (ΔG_binding_ of compound **M3** = −10.0 kcal/mol). In all cases considered, the potency of binding to the active center of protein is similar, and the binding mode of compounds influences their selectivity.

**Figure 4:**
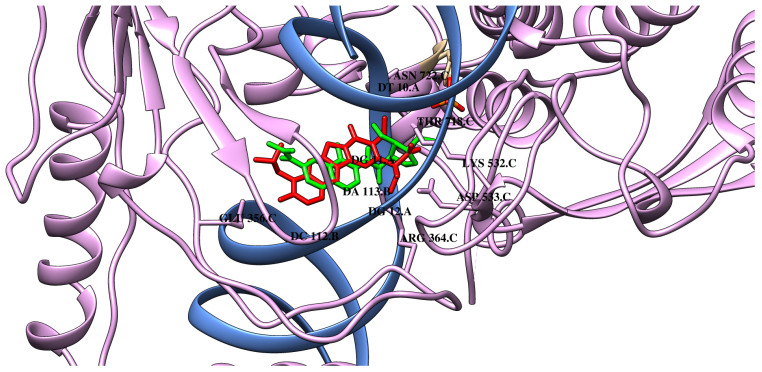
The binding poses of **M0** (red) and **M3** (green) in the topoisomerase I–DNA complex.

The binding mode of compound **M0** is presented in Figure 4 and Figure S4. The obtained data demonstrated that the reference compound (**M0**) engages in multiple hydrogen bonds with the residues ARG364, LYS532, GLU356, and ASP533 (See Table 1). Additionally, the stabilization of the ligand structure within the pocket is facilitated by numerous van der Waals interactions, which also involve nucleic acid residues (DG12, ARG488, PTR723, ASN722, THR718). This is reflected in the interaction analysis, where the proposed ligands preferentially form interactions with residues ARG364, ARG488, PTR723, and ASN722, primarily through van der Waals interactions. Hydrogen bonds are formed between the dimethoxycyclopentane moiety of the ligands and the LYS532 residue, as well as with DG11 through the oxygen atom derived from the amide bond.

As presented in Table 1, compound **M1** has a similar mode of binding. Like many TOPO-I inhibitors, it binds to the hydrophobic pocket, interacting via π-type forces with DT10, DG11, DA113, and DC112, as well as stacks between chains A and B of DNA. Many inhibitors bind to the hydrophobic pocket in a planar configuration due to the aromatic rings that can stack with DNA bases within the cleavage complex, thereby stabilizing the interaction. The same interactions are also observed in the case of compounds **M2**–**M6** and **M8**. Compound **M2** is mainly stabilized in the protein binding pocket by van der Waals interactions with ARG364, ARG488, PTR723, THR718, ASP533, and ILE535. As presented, similar to the **M0** compound, **M2** may create one carbon–hydrogen bond with DT10. As expected, the quinoline scaffold of **M3** intercalates between the cleaved DNA ends within the active site of topoisomerase I. This is a typical interaction for known topoisomerase I inhibitors and also for our reference ligand (**M0**). There are also observed several π-type reactions with the nucleic acid bases, which include DT10, DG11, DC112, and DA113. Additionally, two hydrogen bonds are created in the cleavage region between the oxygen atoms of the inhibitor, as well as DG11 and DA113. As is well known, this positioning can prevent the religation of DNA. Although **M3** does not form hydrogen bonds with conserved amino acid residues such as tyrosine and asparagine, van der Waals interactions stabilize the system in this region. A hydrogen bond is also formed with the LYS532 residue. A similar hydrogen bonding pattern is exhibited by **M4** and **M5** compounds, which are analogously positioned in the binding pocket. The next analyzed compound **M6** is stabilised in the macromolecule by van der Waals interactions with amino acid residues (ARG364, ARG488, PTR723, ASN722, THR718, HIS632) and nucleic acid residue DG12. Similar to **M0**, a hydrogen bond with LYS532 and a carbon–hydrogen bond with DT10 were created. There are also observed π-type interactions in the hydrophobic pocket. In the case of compound **M7**, no hydrogen bonds are observed with amino acid residues, but only with nucleic acids DG11 and DA113. However, analogously to **M0**, a carbon–hydrogen bond is present with residue DT10. Furthermore, π-type interactions are also similar to the case of compound **M0**. **M8** interacts with the topoisomerase I molecule similarly to **M7**. An additional hydrogen bond with the LYS532 residue is observed analogous to the reference compound **M0**. It also forms more carbon–hydrogen interactions than **M0**. In the case of **M9**, additional hydrogen bonds are formed, namely a conventional hydrogen bond with LYS751 and a carbon–hydrogen bond with crucial aspartic acid ASP533. The interactions with the negatively charged phosphate group of DG11 are also present. Compounds **M10**, **M11**, and **M12** have been shown to form van der Waals interactions with ARG488, ASN722, THR718, and PTR723, suggesting a conserved interaction pattern like **M0**. In addition, **M10** and **M12** interact with ASP533, while **M12** forms an additional contact with ASN532 and GLU356, which may contribute to enhanced stabilisation. **M10**, **M11**, and **M12** form hydrogen bonds with LYS532, while **M11** also interacts with ARG364. The π–π interactions with DG11, DA113, and DC112 were also noted for **M10**–**M12**, suggesting a significant contribution to their binding affinity by aromatic ring stacking. All three compounds form alkyl interactions with DT10 and DA113, reinforcing hydrophobic stabilisation within the binding pocket. In addition, **M11** was observed to associate with DG11, suggesting increased affinity by establishing additional hydrophobic contacts. The remaining data are shown in Table 1 and Table 2. Detailed data concerning the interactions of the remaining compounds were presented in Table 1 and Table 2. In Figure 4, the best docking poses of **M0** and **M3** are shown.

**Figure 5:**
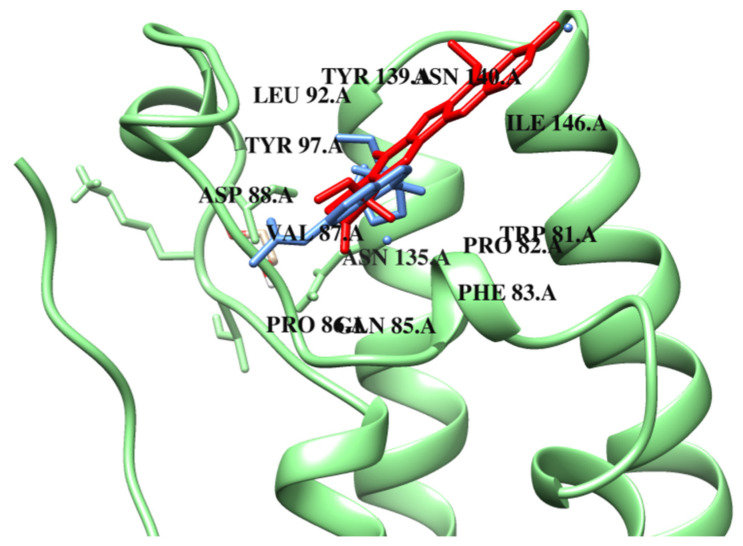
The binding poses of **M0** (red) and **M2** (blue) in BRD4.

Taking into account the molecular docking results, all compounds exhibit the same or better binding ability to BRD4. The free energy of binding for complexes with BRD4 ranges from −6.6 kcal/mol to −8.0 kcal/mol (ΔG_binding_ of compound **M2** = −8.0 kcal/mol). They also show a similar way of binding. The data suggest that a significant number of van der Waals interactions are formed by the designed compounds, which contribute to the stabilization of the ligands within the protein pocket. The binding mode of compound **M0** is presented in Figure 5 and Figure S5. The reference compound, similar to known BDR4 inhibitors, can bind to the acetyl–lysine recognition pocket and can form a hydrogen bond with the conserved asparagine residue. As presented in Table 3, a compound **M0** is involved in a π–alkyl interaction with LEU92. The inhibitor is also stabilized through additional hydrogen bonds with TRP81 and GLN85 and through van der Waals interactions in the hydrophobic cavity of the protein.

As can be seen in Table 3, **M0** shows limited interactions with the protein in contrast to designed compounds that interact with multiple amino acid residues, which may suggest a potential increase in their binding strength and stability of the resulting complexes. Compound **M1** forms many van der Waals interactions, e.g., with ASP88, LEU96, PHE83, and TYR139. Additionally, hydrogen bonds with PRO82 and ASN140 are also possible. According to molecular docking results, compound **M2** might be the best inhibitor of BRD4. Similar to **M1, M2** is involved in π-type interactions with ILE146, LEU92, and VAL87. The system is also stabilized by van der Waals contacts with TRP81, GLN85, PRO86, and LEU94. The comparison of the mode of binding both **M0** and **M2** is presented in Figure 5. Compound M3 forms a hydrogen bond with the same conserved asparagine residue within the binding pocket and engages in hydrophobic interactions similar to JQ1 and I-BET762. It is possible due to the structural elements that enhance its affinity for the bromodomains by extending into adjacent regions of the pocket. As can be observed, the dominant stabilizing factors are van der Waals interactions, with the pocket created by PHE83, LEU92, LEU94, TYR97, ASN135, TYR139, and ASN140. As demonstrated in the present study, compound **M4** exhibits a lower tendency to form van der Waals interactions in comparison to compounds **M1** and **M3** with amino acid residues such as PHE83, PRO86, TYR97, TYR139, and CYS136. The PRO82 amino acid residue has been observed to form hydrogen bonds and carbon–hydrogen bonds, as well as to participate in π–alky interactions with the described compound. Compound **M5** demonstrates a very similar mode of binding to **M2**. In the case of compound **M6**, similarly to those described earlier, several van der Waals interactions are observed. However, hydrogen bonds and carbon–hydrogen interactions are not formed here. Similarly to the reference compound **M0**, a π-type interaction with the ILE3 residue is present. In addition, amino acids VAL87, CYS136, and PRO82 are involved in the π–alkyl interactions. **M7** exhibits a similar protein-binding pattern to that of **M6**. However, in this case, two hydrogen bonds are present with amino acid residues ASN140 and ASP45. A less preferred binding mode is represented by the **M8** and **M9** derivatives. They lack key aspartic acid interactions, and the inhibitors interact directly with the surrounding protein solvent. Compounds **M10**, **M11**, and **M12** show different interactions with BRD4. **M10** interacts via van der Waals forces with residues ASP88, TYR139, ASN140, MET132, TRP81, and PRO86. It also forms hydrogen bonds with TYR97 and PRO82. The carbon–hydrogen interaction occurs via ASN135 and GLN85. **M11** shows a similar van der Waals interaction profile involving ASP88, TYR139, LEU94, and ASN at positions 135 and 140. In addition, **M11** is anchored to BRD4 by carbon–hydrogen interactions (TRP81, TYR97, and PRO86) and π-donor alkyl contacts (ILE146, VAL87, CYS136, and PRO82). Numerous van der Waals interactions were also observed for the binding of **M11**, showing carbon–hydrogen through the following amino acids: TRP81, TYR97, and PRO86. Complex **M11** with BRD4 is also stabilised by π stacking with PHE83 and CYS136. Compound **M12** binds to BRD4 mainly by van der Waals forces (PRO86, PHE83, TYR139, LEU94, ASN140, and GLN84) and π–alkyl interactions through the same amino acids as **M11**. Hydrogen bonds in the protein cavity involving residues ASP88, TYR97, and TRP81 are also important in stabilising the structure. Structures **M10**, **M11**, and **M12** show a greater number of interactions than **M0**, suggesting potentially stronger binding to BRD4. The remaining data are presented in Table 3 and Table 4.

**Figure 6:**
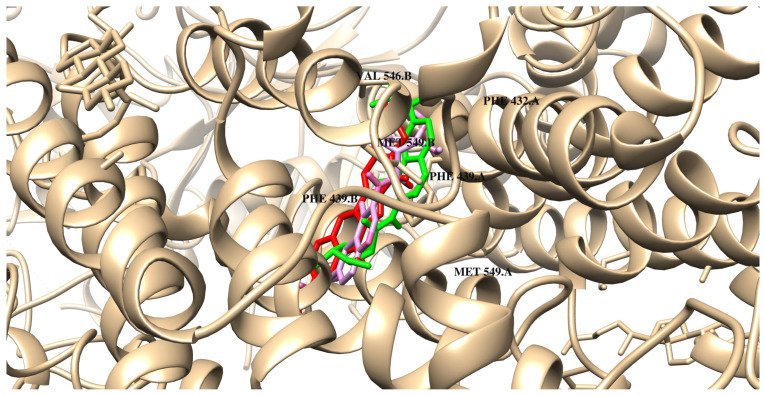
The binding poses of **M0** (red), **M3** (green), and **M12** (pink) in ABCG2.

In the case of interactions with ABCG2, the free energy of binding varies from −8.0 kcal/mol to −9.2 kcal/mol (ΔG_binding_ of compounds **M3** and **M12** = −9.2 kcal/mol). Results revealed that the heterocyclic rings of irinotecan and the designed compounds are primarily responsible for forming interactions with the protein’s amino acid residues. The ligands are localized near the residues PHE439, MET549, and VAL546. These orientations are primarily stabilized by π–π interactions. The quinoline ring in the designed compounds plays a significant role in facilitating interactions with the protein. As presented in Figure 6 and Figure S6 and Table 5, compound **M0** binds to the transmembrane region of ABCG2 and interacts with key amino acid residues. The binding mechanism involves π stacking and π–alkyl interactions with PHE439 and VAL546, respectively. One hydrogen bond is also formed with ASN436. Compounds **M1** and **M3** exhibit very similar modes of binding. As presented in Table 5, both compounds also form one hydrogen bond with ASN436 and interact via π-type stacking forces with PHE439 residues from chain A and B, respectively. According to the data obtained, their quinoline ring interacts with π-type interactions with PHE439, MET549, and VAL546. Another important stabilizing factor of the system is van der Waals interactions with polar amino acids. Compounds **M2**, **M4**, and **M5** are able to form one conventional hydrogen bond with MET549 and a carbon–hydrogen bond with ASN436. Similarly to **M0**, the systems are also stabilized by π-type stacking interactions with PHE439 of chains A and B of the protein. In the case of **M6**, no carbon–hydrogen interaction is observed. π and π–alkyl interactions are present, similar to those in the system with compound **M0**. In the case of the next two compounds, **M7** and **M8**, van der Waals interactions or pi interactions are similar to those of the reference compound. **M8** does not form π–alkyl interactions with MET5499 and PHE439 residues. Both compounds also do not exhibit any hydrogen bonds with the tested protein. Compound **M9** exhibits a very similar mode of binding (Table 6). Numerous van der Waals forces and π–alkyl interactions dominate the binding mode for compounds **M10**, **M11**, and **M12**. These quinolones are stabilised in the binding pocket of ABCG2 by π–π stacking engagement through the phenyl ring: PHE439, both from chain A and B. Furthermore, the sulphur atoms (MET549) are engaged in a forming complex with **M10** and **M12**, similar to **M0**. All detailed interactions are listed in Table 5 and Table 6.

### 2.3. Molecular Dynamics Simulation Results

A 100 ns molecular dynamics simulation was performed for the reference compound (**M0**) and all proposed compounds (**M1**–**M12**). For all systems, from MD complex trajectories, the binding free energy was calculated. The MMPBSA method was used. The results are presented in Table 7. For all complexes, the ΔGbind is negative, indicating spontaneous complex formation. For the interaction with topoisomerase I–DNA, the binding free energy was calculated between −15.01 ± 2.67 kcal/mol and −28.73 ± 1.26 kcal/mol. For all systems, except with **M2**, the values are more negative than for the system with the reference drug. The best results were found for complexes with **M3** and **M11**. For the interaction with the bromodomain-containing protein 4 (BRD4), the most negative energy was found for systems with **M3** (−25.92 ± 3.71 kcal/mol) and **M12** (−25.06 ± 2.61 kcal/mol). These values are better than for the system with the reference drug **M0** (−22.21 ± 2.69 kcal/mol). For the interaction with the ATP-binding cassette sub-family G member 2 protein (ABCG2) for all systems, the ΔG_bind_ is more negative than for the system with the reference drug. The best values were found for **M9** (−43.97 ± 1.83kcal/mol), **M4** (−38.99 ± 2.22 kcal/mol), and **M12** (−38.89 ± 2.09 kacl/mol). These results indicate that the studied compounds have the potential to be good inhibitor candidates.

**Figure 7:**
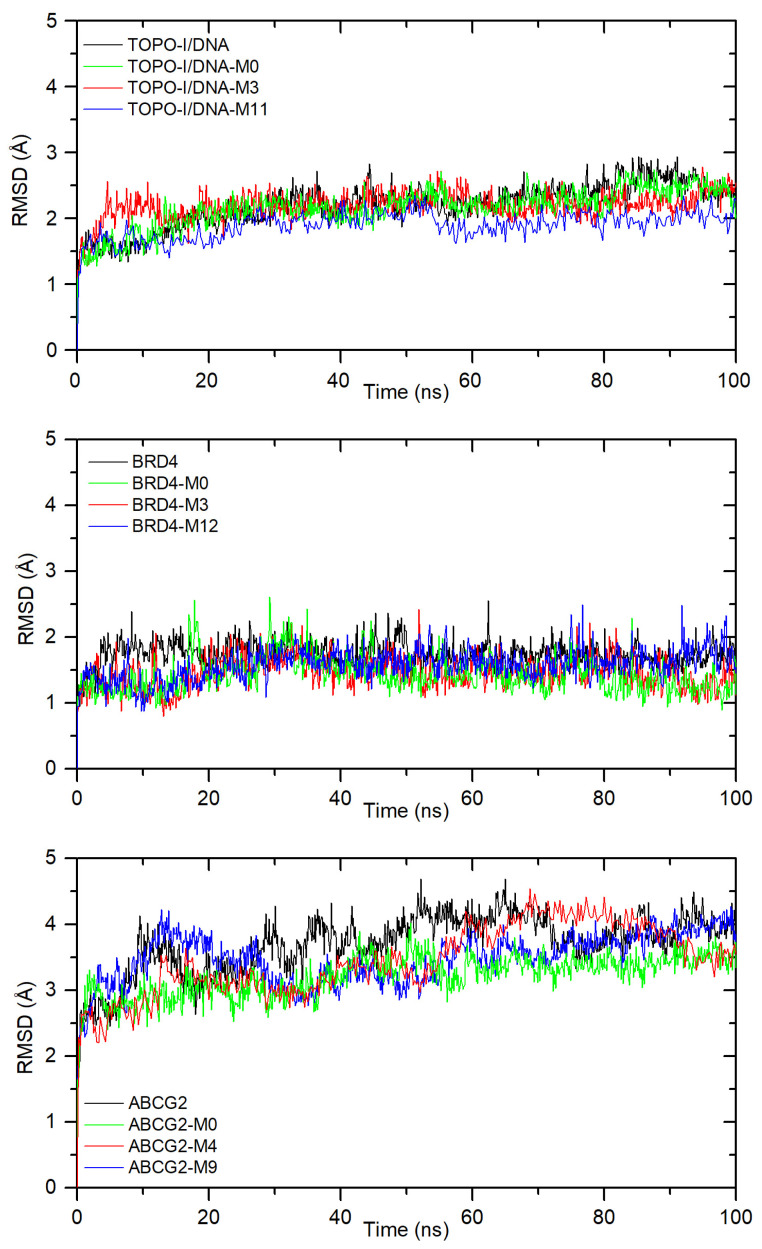
The RMSD plot of the protein backbone after least-squares fitting to the protein backbone for complexes with TOPO-I/DNA, BRD4, and ABCG2.

Figure 7 shows the RMSD plot of the protein backbone after the least-squares fitting to the protein backbone, for free protein and all systems with compounds, the most negative is ΔG_bind_. All complexes quickly stabilize with small fluctuations during the simulation (0.3–0.6 Å). The averaged RMSD was found to be: 2.20 ± 0.33 Å, 2.15 ± 0.30 Å, 2.19 ± 0.22 Å, and 1.89 ± 0.22 Å for free TOPO-I/DNA, and complexes with **M0**, **M3**, and **M11**, respectively. For systems with BRD4, the RMSD was calculated to be 1.75 ± 0.19 Å (without ligand), 1.14 ± 0.25 Å (with **M0**), 1.45 ± 0.24 Å (with **M3**), and 1.58 ± 0.24 Å (with **M12**). The highest fluctuations are observed for systems with the large ABCG2 protein. The RMSD was calculated as 3.70 ± 0.46 Å (without ligand), 3.21 ± 0.31 Å (with **M0**), 3.45 ± 0.54 Å (with **M4**), and 3.48 ± 0.39 Å (with **M9**). After binding into a complex, for all proteins, the RMSD values are lower than for the protein without a ligand. This suggests greater rigidity in the ligand presence. Similar results were obtained for the other complexes. All systems stabilize during the simulation, although some fluctuations occur. The RMSD plots are shown in Figure S7 in the Supplementary File.

**Figure 8:**
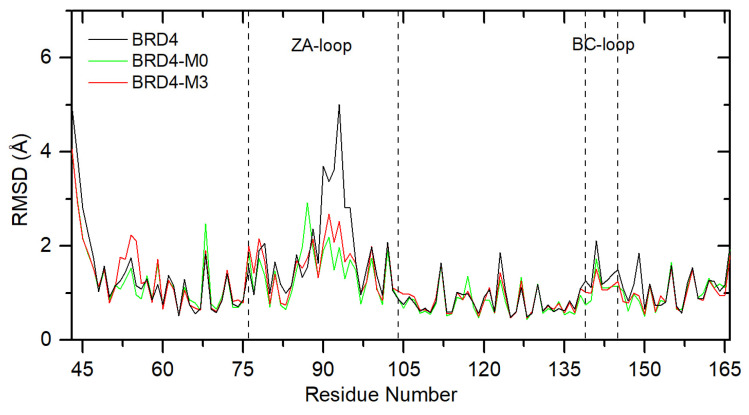
The RMSF plot of the BRD4 protein residue without ligand and with **M0** and **M3**.

The time-average RMSF for each residue was calculated for analysis of the local changes in protein structure. Figure 8 shows the plot for **M0** and **M3**, complexes with BRD4. For the free protein, the most flexible regions are the ZA loop and the BC loop. The sum of the RMSF value for all BRD4 residues was calculated as 161.1 Å. After ligand **M3** binding, the sum of RMSF decreases to 146.7 Å (8.9%). The same was observed for the reference compound (139.5 Å, 13.4%). Even greater differences were found for the ZA loop, 54.9 Å (free), 45.4 Å (**M3**, 17.3%), 41.8 Å (**M0**, 23.9%), and the BC loop 10.9 Å (free), 9.1 Å (**M3**, 16.5%), and 8.8 Å (**M0**, 19.2%). These results indicate an increase in protein rigidity and greater structural stability upon ligand binding. A similar effect was also observed for the other two proteins. The rigidity of the protein increases by 7.9% (for TOPO-I/DNA) and 7.2% (for ABCG2) in the presence of **M3**, as well as 3.3% (for TOPO-I/DNA) and 3.9% (for ABCG2) in the presence of **M0**. A similar effect occurred for the other ligands.

### 2.4. ADMET Properties of the Tested Compounds

One of the primary objectives of optimal drug design is to achieve precise matching of pharmacodynamic, pharmacokinetic, and ADMET parameters. The determination of these values enables the prediction of the drug’s behavior within the body and the identification of potential obstacles to be overcome. Furthermore, knowledge of these parameters enables the optimal route of administration to be selected. At present, numerous software programs facilitate the prediction of ADMET properties, which has markedly enhanced the overall process. The aforementioned tools facilitate the early exclusion of unsuitable compounds, thereby reducing the number of potential syntheses and subsequent failures. The prediction of ADMET properties in silico constitutes an indispensable aspect of contemporary drug design [18].

The ADMET properties of the designed compounds were determined using the online tool ADMETlab 3.0 [19]. In the first step, we determined the physicochemical profile of the analyzed compounds. The results obtained are summarized in Table 8.

The molecular weight of all designed compounds is within the reference range, as expected. Furthermore, none of the compounds approaches the upper limits, which may indicate an optimal degree of distribution and absorption. The number of hydrogen bond donors and acceptors also meets the assumed conditions. It can, therefore, be concluded that the permeability through biological membranes should not be hindered. The next two parameters, Nrot and PSA, which determine bioavailability and pharmacokinetic parameters, are also within the reference range. The obtained logP values are also satisfactory. The most hydrophilic compound is **M5**, while the most lipophilic is **M9**. However, the parameter describing solubility (logS) is the least favorable. The tested molecules have moderate or poor solubility in water. However, it is worth emphasizing here that all designed compounds have better solubility than the reference compound **M0**. Lipophilicity of the structure: SN-38 is more lipophilic than irinotecan, contributing to its greater cellular uptake and stronger interaction with DNA. This lipophilicity also plays a role in enhancing the interaction with the enzyme–DNA complex within the hydrophobic environment of the replication machinery. It is noteworthy that all the tested compounds comply with Lipinski’s Rule of 5 [20]. The swissADME tool was utilized to calculate the synthetic accessibility score, which is indicative of the hypothetical difficulty of synthesizing the studied compounds. The values calculated for the **M1**–**M12** molecules are listed in Table 8. For all designed compounds, this value is below 4, indicating that synthesis should not be difficult. However, it is imperative to acknowledge the presence of three chiral centers in these compounds, necessitating the employment of an appropriate strategy during synthesis to achieve the desired stereoisomers of each compound under analysis. Stereoselective synthesis can be achieved through three distinct mechanisms: substrate control using a chiral auxiliary, reagent control using a chiral reagent, or the utilization of a chiral catalyst. An alternative approach involves absolute asymmetric synthesis, which entails spontaneous resolution [21]. In the previously published works on the synthesis of cyclopentanes, various strategies have been used, including rhodium-catalyzed domino sequence, cycloaddition reactions, cyclopropane rearrangements, or cyclopentane ring annulation [22,23,24,25]. It should be emphasized that the synthesis of this type of compound requires precise stereochemical control and optimized reaction conditions for high efficiency. The next step was to determine the absorption, and distribution parameters were determined (Table 9). Caco-2 (Colon Adenocarcinoma Cells 2) and MDCK (Madin–Darby Canine Kidney cells) cell culture models are highly effective in simulating drug absorption. The primary factor influencing this process is solubility, which can be used to predict the transport of the drug from the intestine to the bloodstream [26,27]. In the case of Caco-2, the application of this calculation enables the assessment of the extent of intestinal absorption of a molecule to determine whether the transport of a particular compound is active or passive, and to predict the degree of intestinal metabolism. The reference range has been established at values exceeding −5.15. It has been demonstrated that within this range, the degree of intestinal absorption exhibits a variability of approximately 50 to 100% [26,27]. The obtained values for all tested compounds (**M1**–**M12**) fall within the reference range. In the case of the initial structure (**M0**), the resulting value is nearly equivalent to the threshold limit.

In 1999, Jennifer Irvine and coworkers proved that MDCK cells are a good tool for the assessment of the permeability properties in drug discovery [28]. We also decided to determine MDCK values in our research (Table 9). This parameter is particularly pertinent to the processes of renal transport and the crossing of the blood–brain barrier. A compound is deemed to have a low permeability if its value is less than 2 × 10^−6^ cm/s. A permeability range of 2–20 × 10^–6^ cm/s is indicative of compounds with medium permeability. Those with high permeability are characterized by values exceeding 20 × 10^−6^ cm/s [27]. It can be predicted that all newly designed compounds, as well as the initial structure, will exhibit low permeability since they assume negative values.

P-glycoprotein is a transmembrane protein that utilizes the energy from ATP to facilitate the movement of molecules across cell membranes. It is responsible for regulating the permeability of the cell membrane to drugs, thereby controlling the movement of substances in and out of the cell. It serves to prevent the excessive accumulation of compounds within the cell. This protein is present in the membranes of lymphocytes and the endothelium of capillaries that form the blood–brain barrier, as well as in the intestines, kidneys, placenta, and liver. The activity of P-glycoprotein has an impact on the ADMET parameters of drugs. Some compounds can inhibit this membrane protein. The inhibition of P-glycoprotein may result in the accumulation of the compound within the cell, potentially leading to the release of toxic effects [29]. Additionally, some substances are substrates for this protein [30]. Consequently, this results in the accelerated elimination of the drug, which may ultimately lead to the failure to achieve the desired therapeutic concentrations. It is, therefore, crucial to be aware of the effects that a particular drug may have on P-glycoprotein. Awareness of this information enables the avoidance of unfavorable drug combinations and the establishment of dosing regimens that will be most beneficial to the patient. Among the newly designed compounds, those with the highest propensity to inhibit P-glycoprotein are compounds **M8** and **M10** (Table 9). The majority of these compounds cannot inhibit this protein. It can be reasonably deduced that the **M12** compound and the initial structure, designated **M0**, are the most probable substrates of P-glycoprotein.

**Figure 9:**
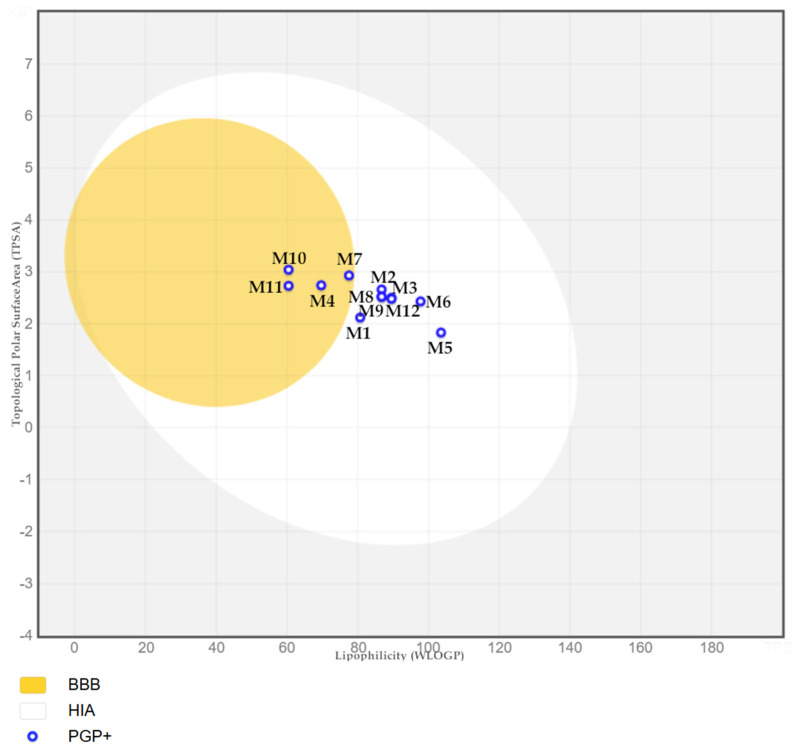
The heuristic model of the boiled-Egg plot, create by SwissAdme [31].

To foresee gastrointestinal absorption and brain penetration of the studied molecules, the boiled-egg plot was created (Figure 9). **M4**, **M7**, **M10**, and **M11** compounds are found in the yolk. There is a high probability of these permeating the BBB (blood–brain barrier). This would allow them to easily access the CNS (central nervous system). All other compounds are contained within a white ellipse, representing an egg white. This indicates that they are likely to be passively absorbed by the gastrointestinal tract. It is predicted that all molecules will be P-glycoprotein (PGP+) substrates, meaning they will be actively pumped out of the brain or into the gastrointestinal lumen.

The next ADMET parameter we evaluated is the degree of binding to plasma proteins. This is one of the most important parameters determining the distribution of a drug in the body. Binding a molecule to a plasma protein, e.g., albumin, causes the drug to have no pharmacological activity. Moreover, in this state, it is not able to penetrate cells, is not metabolized, and is not subject to excretion processes. The higher the degree of binding to proteins, the smaller the percentage of the administered dose that affects the body. On the other hand, the drug–plasma protein complex is a specific drug storage from which the molecule can be released. In the case of the compounds under investigation, the highest degree of protein binding is demonstrated by the initial structure **M0**, which reaches a value of 98.89%. Furthermore, the compounds **M6**–**M11** also demonstrate a high degree of binding to plasma proteins. The lowest percentage of binding is exhibited by compound **M1**, which is only 61.4%. The high degree of plasma protein binding represents a challenge in conducting appropriate pharmacotherapy. However, it does not preclude further investigation of a particular compound’s structure.

The biological half life of the newly designed compounds is quite short, but this is not a reason to exclude the molecule from further research (see Table S1 in Supplementary Files). Current technologies make it possible to modify this parameter. For example, the administration of a molecule in prodrug form or the development of a suitable extended-release formulation can extend t1/2. Additionally, structural modification as halogenation, can significantly influence the half life of drugs by altering their metabolic stability, solubility, and interaction with metabolic enzymes. Therefore, it will be interesting to discuss in the future the effect of the substitution of the quinoline ring with halogens on the potential activity of our compounds.

The CYP2C19 isoenzyme is involved in the metabolism of drugs such as proton pump inhibitors (now very common), some antidepressants, and others. Potential inhibitors are compounds **M9** and **M11**. Both compounds may interact with the above drugs and interfere with their metabolism. Substrates for CYP2C19 are **M1**, **M3**, **M4**, **M10**, **M11**, and **M12** (see Table S2 in Supplementary Files). Another isoenzyme is CYP2D6. It is involved in the metabolism of TLPDs (tricyclic antidepressants), neuroleptics, opioids, or b-blockers. B-adrenoceptor blockers are commonly used drugs due to the increasing number of people suffering from cardiovascular problems. Opioids, antidepressants, or neuroleptics are the most commonly used drugs in cancer pharmacotherapy. It is, therefore, important to know the effects of these molecules on this isoenzyme. However, when analyzing the results presented here, it can be concluded that none of the molecules show the ability to inhibit CYP2D6. Furthermore, only compound **M10** appears to be a substrate for this enzyme. The CYP3A4 isoenzyme is the most common drug-metabolizing enzyme. There are a large number of drugs that are metabolized by it to a greater or lesser extent. There are also many inhibitors and inducers of this enzyme. Therefore, information on the effects of molecules on this isoenzyme is important. As presented in Table S2 in Supplementary Files, the inhibitors of CYP3A4 are the reference compounds **M0**, **M3**, **M4**, and **M10** quinoline derivatives. In contrast, the compounds that are metabolized by this isoenzyme are **M1**, **M3**, **M4**, **M9**, **M10**, and **M11**.

Anticancer therapy is an aggressive procedure, so it is difficult to avoid the side effects. However, it is important to try to minimize them. The results collected in Table S3 in Supplementary Files show that this goal has been partially achieved. Most of the properties shown are reduced compared to the reference compound, and, in some cases, are minimized to zero. Furthermore, none of the compounds, **M1**–**M12**, are more likely to induce the evaluated toxicity parameters.

## 3. Materials and Methods

### 3.1. Molecular Docking

In this study, we choose three molecular targets. Their structures were downloaded from the Nucleic Acid Database [32]. The first is a structure of the complex of human topoisomerase I with camptothecin and DNA, designated as 1RR8 [33]. Another molecular target is the BRD4 subunit in complex with a benzodiazepine derivative as an inhibitor and 1,2-ethanediol (labelled 6CD4 in the base) [34]. The last protein is a complex of the ABCG2 protein with the SN-38 inhibitor designated as 6VXJ [1]. All the tested compounds were optimized at the 6–31++G** level of theory, taking into account the solvent effect (polarizable continuum model, PCM) by using the Gaussian 09 package (Gaussian Headquarters, Wallingford, CT, USA) [35,36]. To predict the binding mode of compounds into the binding site of topoisomerase, the AutoDock 4.2 package with a standard protocol was used [17]. Validation of the docking procedure was performed by docking co-crystalized ligands (camptothecin, benzodiazepine derivative, and SN-38) into the crystal structures of the molecular targets and comparing their position in the original crystallographic structures. The root mean-square deviation (RMSD) was calculated to measure the docking prediction accuracy on the LigRMSD 1.0 web server [37]. The RMSD values were established on 0.95 Å, 1.47 Å, and 1.27 Å for topoisomerase I, BRD4, and ABCG2, respectively. The grid box size for topoisomerase I was set to 50 × 50 × 50 Å with a spacing of 0.375 Å, centered at the coordinates: x = 21.281; y = −3.337; z = 28.559. For the BRD4 protein, the same grid box dimensions were maintained, with the center positioned at: x = 15.039; y = −10.000; z = −1.678. The box size was also replicated for ABCG2, with the docking center set to x = −0.441; y = −0.117; z = 3.558. The obtained results were visualized using a Chimera 1.19 and a BIOVIA Discovery Studio visualizer (Dassault Systèmes Corporate, Dassault Systèmas, Waltham, MA, USA, https://discover.3ds.com/ accessed on 9 April 2025) [38].

### 3.2. Molecular Dynamics Simulation Details

Molecular dynamics (MD) simulations were performed using GROMACS version 2021.2. The starting point was the position of the ligands with the best binding affinity (docking studies). Simulations were conducted for TOPO-I/DNA, BRD4, and ABCG2 systems with all proposed derivatives, as well as with the reference compound **M0**.

System preparation for the MD simulations was conducted using the web-based CHARMM-GUI platform (https://www.charmm-gui.org/ accessed on 9 April 2025) [39,40]. The initial docking results provided the orientations of the ligands in their respective active sites, and the ligand topology files were generated using the CHARMM General Force Field (CGenFF) version 2.5.1 [41]. The protonation states of acidic and basic residues were assigned based on the local environment at pH 7. All complexes were solvated in a rectangular water box with an edge distance of 10 Å, and the systems were further solvated using the TIP3P water model [42]. Sodium and chloride ions were added at a concentration of 0.15 M to neutralize the system’s net charge, with Na^+^ and Cl^−^ ions placed in positions of suitable electrostatic potential by replacing water molecules.

Energy minimization was conducted using the steepest descent algorithm with a tolerance of 1000 kJ/mol·nm. Once the systems converged, MD simulations were performed using the NVT ensemble for 12,500 steps. Following this, NPT ensemble simulations were conducted under periodic boundary conditions, with the CHARMM36m force field applied to the protein. Temperature and pressure were maintained at 303.15 K and 1 bar using the Berendsen thermostat and barostat, respectively. The MD simulations were run for a total of 100 ns under constant pressure and temperature conditions.

The binding free-energy calculations were performed using GMX_MMPBSA (https://www.charmm-gui.org/ accessed on 9 April 2025) [43]. The plots were made using OriginPro 2024 v.10.1

### 3.3. ADMET Evaluation

The ADMET properties of designed compounds were determined using the online tool ADMETlab 3.0 from [https://admetlab3.scbdd.com accessed on 12 May 2025] [19]. The SAScore was evaluated in the SwissADME web tool (http://www.swissadme.ch accessed on 9 April 2025/) [31].

## 4. Conclusions and Summary

In conclusion, our study on the designed quinoline derivatives demonstrates their potential as multifunctional anticancer agents with the ability to bind to multiple molecular targets. It was established that all compounds exhibit satisfactory binding energies and bind in a typical inhibitor manner, suggesting strong interactions with their respective targets. In addition, all compounds exhibit satisfactory drug-like properties, including favorable physicochemical characteristics and transport profiles. Our analyses indicate that these compounds meet essential criteria for solubility, stability, and permeability, supporting their potential for effective bioavailability and distribution. To summarize, the promising results support their therapeutic potential.

The rightness of our strategy to modify the structure of 7-ethyl-10-hydroxycamptothecin, leading to the design of new potential multi-target anticancer drugs, is confirmed by the following results:

- All designed compounds bind to the active site of the three tested proteins with negative binding affinity;
- Molecular dynamics simulation confirmed the stability of the formed complexes, and the calculated binding free energies for all derivatives were found to be negative (Table 7);
- Reducing the size of the molecule does not affect key structural elements that determine binding to selected molecular targets;
- The introduced modifications had a positive impact on the ADMET parameter profile (Table 9);
- Designed compounds have favorable physicochemical properties and relatively low toxicity (Table 8 and Table S3);
- Potential disadvantages, such as a short biological half life or effects on metabolic enzymes, can be overcome, for example, by developing a suitable formulation of the new drug.

Our results strengthen the case for further development and highlight the potential of these compounds as promising candidates for anticancer therapy. It is important to note that the compounds designed are stereoisomers that may have different biological and chemical properties. Stereoisomers often differ in pharmacological activity and toxicity, and they may target different metabolic pathways in the body. Therefore, great care must be taken in their synthesis as it is crucial to obtain the desired stereoisomer with high purity.

It should be underlined that, in this study, we have focused on preliminary studies by in silico methods. However, this is only the first step in the design of new drugs, which can save time and costs in subsequent stages of research. The next step of the project should focus on an experimental study and potential implementation. All of this is a real challenge and is fraught with risk to the success of the application of the new drug. Multi-target compounds may interact with unintended targets, which can lead to harmful side effects. These interactions can be difficult to predict using in silico methods, especially when they involve rare or poorly understood biological pathways. Identifying reliable biomarkers to stratify patients and monitor response to treatment can be challenging, especially when multiple pathways are targeted. Recent limitations include the fact that the approval process for multi-target inhibitors is often more complicated than for single-target drugs due to concerns about safety, efficacy, and side effects. The multi-target nature of these compounds requires more data, which may delay or prevent approval and complicate the path to clinical use.

## Figures and Tables

**Table 1:** Interactions of compounds **M0**–**M6** in the binding cavity of the topoisomerase I.

	M0	M1	M2	M3	M4	M5	M6
**van der Waals interactions**	DG A:12 ARG C:488 PTR C:723 ASN C:722 THR C:718	ARG C:488 PTR C:723 ASN C:722 ILE C:535	ARG C:364 DG A:12 ARG C:488 PTR C:723 THR C:718 ASP C:533 ILE C:535	ARG C:364 DG A:12 ARG C:488 ASN C:722 THR C:718 LYS C:452	ARG C:364 DG A:12 ARG C:488 PTR C:723 ASN C:722 THR C:718	ARG C:364 DG A:12 ARG C:488 PTR C:723 ASN C:722 THR C:718	ARG C:364 DG A:12 ARG C:488 PTR C:723 ASN C:722 THR C:718 HIS C:632
**Hydrogen bonds**	ARG C:364 LYS C:532 GLU C:356 ASP C:533	DG A:11 ARG C:364 THR C:718	DG A:11 DA B:113 LYS C:532	DG A:11 DA B:113 LYS C:532	DG A:11 LYS C:532	DG A:11 LYS C:532	DG A:11 DA B:113 LYS C:532
**Carbon–hydrogen interactions**	DT A:10	DG A:11 ASP C:533 HIS C:632 DG A:12	DT A:10	DT A:10 DG A:11	DT A:10 DG A:11 ASN C:532	DT A:10 DG A:11 ASP C:533	DT A:10 DG A:11
**π-type interactions**	DA B:113 DG A:11 DC B:112	DT A:10 DG A:11 DA B:113 DC B:112	DT A:10 DG A:11 DA B:113 DC B:112	DT A:10 DG A:11 DA B:113 DC B:112	DG A:11 DA B:113 DC B:112 DT A:10	DT A:10 DG A:11 DA B:113 DC B:112	DT A:10 DG A:11 DA B:113 DC B:112
**Alkyl/π** **–alkyl interactions**	DG A:11	DA B:113	DT A:10 DG A:11 DA B:113	DT A:10	DT A:10	DT A:10	DT A:10 DA B:113

**Table 2:** Interactions of compounds **M7**–**M12** in the binding cavity of topoisomerase I.

	M7	M8	M9	M10	M11	M12
**van der Waals interactions**	ARG C:364 DG A:12 ARG C:488 PTR C:723 ASN C:722 THR C:718 LYS C:532	ARG C:364 DG A:12 ARG C:488 PTR C:723 ASN C:722 THR C:718 ASP C:533 HIS C:632	DG A:12 ARG C:488 PTR C:723 ASN C:722 THR C:718	ARG C:364 DG A:12 ARG C:488 PTR C:723 ASN C:722 THR C:718 ASP C:533	ARG C:488 ASN C:722 THR C:718	ARG C:488 PTR C:723 ASN C:722 THR C:718 ASP C:533 ASN C:532 GLU C:356
**Hydrogen bonds**	DG A:11 DA B:113	DG A:11 DA B:113 LYS C:532	DG A:11 LYS C:532 LYS C:751 DT A:10	DG A:11 LYS C:532	ARG C:364 LYS C:532	DG A:11 LYS C:532
**Carbon–hydrogen interactions**	DT A:10 ASP C:533	DT A:10 DG A:11 GLU C:356	ASP C:533 DA B:113 DA B:114	DT A:10	PTR C:723	DT A:10 DG A:11
**π-type interactions**	DG A:11 DA B:113 DC B:112	DT A:10 DG A:11 DA B:113 DC B:112	DT A:11 DT A:10	DG A:11 DA B:113 DC B:112	DA B:113 DG A:11 DC B:112	DA B:113 DG A:11 DC B:112
**π** **–alkyl/alkyl**	DT A:10 DA B:113	DT A:10 DA B:113	LEU C:721	DT A:10 DA B:113	DT A:10 DG A:11 DA B:113	DT A:10 DA B:113

**Table 3:** Interactions of compounds **M0**–**M6** with BRD4.

	M0	M1	M2	M3	M4	M5	M6
**van der Waals interactions**	ASP A:88 ASP A:144	TRP A:81 LYS A:91 ASP A:88 GLN A:85 PRO A:86 LEU A:96 PHE A:83 TYR A:139 CYS A:136	TRP A:81 GLN A:85 PRO A:86 PHE A:83 TYR A:139 LEU A:94 ASN A:140	TRP A:81 PHE A:83 TYR A:139 LEU A:94 ASN A:140 TYR A:97 ASN A:135 LEU A:92 ASP A:144	PRO A:86 PHE A:83 TYR A:139 CYS A:136 TYR A:97	ASP A:88 GLN A:85 PRO A:86 PHE A:83 TYR A:139 ASN A:140 ASN A:135	TRP A:81 PHE A:83 TYR A:139 LEU A:94 TYR A:97 LEU A:92 ASP A:144
**Hydrogen bond**	ASP A:145 GLN A:85 TRP A:81	PRO A: 82 ASN A:140	ASP A:88 TYR A:97	ILE A:146 ASP A:145	PRO A:82 ASN A:140	TYR A:97	
**Carbon–hydrogen interactions**	VAL A:87	ASN A:140	ASN A:135		PRO A:82 GLN A:85	MET A:132	
**π** **-type interactions**	PRO A:86 ILE A:146	LEU A:92 ILE A:146	ILE A:146	LEU A:92	ILE A:146	LEU A:92 ILE A:146	ILE A:146
**π–alkyl/alkyl**	LEU A:92	VAL A:87 TYR A:97 LEU A:92	LEU A:92 VAL A:87 CYS A:136 PRO A:82	VAL A:87 CYS A:136 PRO A:82	VAL A:87 PRO A:82 TRP A:81	VAL A:87 CYS A:136 PRO A:82 TRP A:81	VAL A:87 CYS A:136 PRO A:82

**Table 4:** Interactions of compounds **M7**–**M12** with BRD4.

	M7	M8	M9	M10	M11	M12
**van der Waals interactions**	TRP A:81 PHE A:83 TYR A:139 LEU A:94 TYR A:97 LEU A:92 ASP A:144	ASP A:88 GLN A:85 PRO A:86 PHE A:83 TYR A:139 ASN A:140	ASP A:88 GLN A:85 PRO A:86 PHE A:83 TYR A:139 LEU A:94 TYR A:97	ASP A:88 PRO A:86 PHE A:83 TYR A:139 ASN A:140 MET A:132 TRP A:81	ASP A:88 TYR A:139 ASN A:140 ASN A:135 LEU A:92	PRO A:86 PHE A:83 TYR A:139 LEU A:94 ASN A:140 GLN A:84
**Hydrogen bonds**	ASN A:140 ASP A:145	TYR A:97		PRO A:82 TYR A:97	GLN A:85	ASP A:88 TYR A:97 TRP A:81
**Carbon–hydrogen interactions**		ASN A:135 MET A:132 TRP A:81	ASN A:140 VAL A:87	ASN A:135 GLN A:85	TRP A:81 TYR A:97 PRO A:86	ASN A:135
**π-type interactions**	ILE A:146				PHE A:83 CYS A:136	LEU A:92
**π–alkyl/alkyl**	ILE A:146 VAL A:87 CYS A:136 PRO A:82	ILE A:146 LEU A:92 VAL A:87 CYS A:136 PRO A:82 TRP A:81	ILE A:146 LEU A:92 VAL A:87 CYS A:136 PRO A:82 TRP A:81	ILE A:146 LEU A:92 VAL A:87 CYS A:136 PRO A:82	ILE A:146 VAL A:87 CYS A:136 PRO A:82	ILE A:146 VAL A:87 CYS A:136 PRO A:82

**Table 5:** Interactions of compounds **M0**–**M6** with ABCG2.

	M0	M1	M2	M3	M4	M5	M6
**van der Waals interactions**	THR B:435 SER A:440 PHE A:432 SER B:440 THR B:542 ASN A:436 MET B:549	LEU B:405 PHE B:432 THR B:435 SER A:440 THR A:435 PHE A:432 SER B:440	LEU B:405 PHE B:432 THR B:435 SER A:440 THR A:435 PHE A:432 SER B:440 ASN B:436	PHE B:432 THR B:435 SER A:440 THR A:435 PHE A::432 SER B:440 THR B:542 ILE B:543	PHE B:432 THR B:435 SER A:440 THR A:435 PHE A:432 SER B:440	PHE B:432 THR B:435 SER A:440 THR A:435 PHE A:432 SER B:440 ASN B:436	SER A:440 THR A:435 PHE A:432 THR B:542 ASN B:436 LEU A:405 VAL B:546
**Hydrogen bonds**	ASN B:436	ASN B:436	MET B:549	ASN A:436	MET A:549	MET B:549	ASN A:436
**Carbon–hydrgen interactions**	THR A:435	ASN A:436	ASN A:436	ASN B:436	ASN A:436 ASN B:436	ASN A:436	
**π-type interactions**	PHE A:439 PHE B:439	PHE A:439 PHE B:439	MET A:549 PHE B:439 PHE A:439	PHE A:439 PHE B:439	PHE A:439 PHE B:439	PHE B:439 PHE B:439	PHE B:439 PHE A:439
**π–alkyl/alkyl**	VAL A:546 VAL B:546 MET A:549 PHE B:432	VAL A:546 VAL B:546 MET A:549 PHE A:439	VAL A:546 VAL B:546 PHE A:439	VAL A:546 VAL B:546 MET B:549 PHE B:439	VAL A:546 VAL B:546 MET B:549 PHE B:439	VAL A:546 VAL B:546 MET A:549 PHE A:439	VAL A:546 MET B:549 PHE A:439
**Sulfur interactions**	MET B:549	MET B:549	MET B:549	MET B:549 MET A:549	MET B:549	MET B:549	MET A:549

**Table 6:** Interactions of compounds **M7**–**M12** with ABCG2.

	M7	M8	M9	M10	M11	M12
**van der Waals interactions**	PHE B:432 THR B:435 SER A:440 THR A:435 PHE A:432 SER B:440 ASN A:436	PHE B:432 THR B:435 SER A:440 THR A:435 SER B:440 ASN A:436 ASN B:436 ILE B:543	PHE B:432 THR A:435 PHE A:432 SER B:440 ASN A:436 ASN B:436 LEU A:405 THR A:435	LEU B:405 THR B:435 THR A:435 SER B:440 ASN A:436	PHE B:432 THR B:432 THR A:435 PHE A:432 ASN A:436 ASN B:436	LEU B:405 PHE B:432 THR B:435 SER A:440 THR A:435 PHE A:432 SER B:440 ILE A:543 VAL B:401
**Hydrogen bonds**						MET B:549 ASN B:436
**Carbon–hydrogen interactions**	ASN B:436	THR B:542	PHE A:439			ASN A:436
**π-type interactions**	PHE A:439 PHE A:439	PHE A:439 PHE A:439 PHE B:439	PHE A:439 PHE A:439 PHE B:439	PHE A:439 PHE B:439	PHE A:439 PHE B:439	PHE A:439 PHE B:439
**π–alkyl/alkyl**	VAL A:546 VAL B:546 MET B:549 PHE B:439	VAL A:546 VAL B:546	VAL B:546 MET A:549 PHE B:439	VAL A:546 MET A:549 MET B:549 PHE A:432 PHE B:432	VAL A:546 VAL B:546 MET A:549 MET B:549 PHE A:439 PHE B:439	VAL A:546 VAL B:546 PHE A:439
**Sulfur interactions**	MET A:549	MET B:549 MET A:549	MET B:549	MET A:549		MET B:549 MET A:549

**Table 7:** The binding free energy for complexes with **M0**–**M12**. Results from molecular dynamics simulation.

Compound	Binding Free Energy (MD) (kcal/mol)		
	TOPO-I/DNA	BRD4	ABCG2
**M0**	−18.19 ± 2.53	−22.21 ± 2.69	−25.42 ± 2.93
**M1**	−19.57 ± 2.52	−21.60 ± 1.78	−30.41 ± 1.93
**M2**	−15.01 ± 2.67	−15.48 ± 1.87	−35.00 ± 3.06
**M3**	−24.98 ± 2.65	−25.92 ± 3.71	−37.62 ± 3.73
**M4**	−21.13 ± 3.23	−17.60 ± 1.86	−38.99 ± 2.22
**M5**	−22.74 ± 1.29	−21.02 ± 2.45	−36.24 ± 1.80
**M6**	−18.97 ± 1.38	−24.67 ± 2.53	−36.05 ± 2.05
**M7**	−24.09 ± 1.05	−23.84 ± 2.63	−37.36 ± 2.11
**M8**	−23.87 ± 1.39	−19.93 ± 2.15	−37.76 ± 1.51
**M9**	−23.63 ± 3.25	−21.28 ± 2.77	−43.97 ± 1.83
**M10**	−19.50 ± 2.24	−22.01 ± 1.94	−34.06 ± 3.06
**M11**	−28.73 ± 1.26	−23.76 ± 2.23	−28.10 ± 2.40
**M12**	−22.67 ± 2.96	−25.06 ± 2.61	−38.89 ± 2.09

**Table 8:** The physicochemical properties of the compounds **M1**–**M12** and the reference structure **M0** were determined using the ADMETlab 3.0 (https://admetlab3.scbdd.com/ accessed on 9 April 2025) program. SAscore was obtained by swissADME tool (http://www.swissadme.ch/ accessed on 9 April 2025).

Property	MW [Da]	HBA	HBD	Nrot	TPSA [Å^2^]	logP	logS	SAscore
**M0**	392.14	7	2	2	101.65	1.045	−3.581	-
**M1**	332.17	6	2	5	80.68	1.027	−2.407	4.27
**M2**	372.17	7	1	7	86.75	1.530	−2.786	3.83
**M3**	371.18	7	2	7	89.55	1.551	−2.775	3.86
**M4**	344.17	6	1	6	69.68	1.935	−3.077	3.70
**M5**	357.17	7	3	6	103.54	0.963	−2.608	3.70
**M6**	358.15	7	2	6	97.75	1.491	−3.109	3.69
**M7**	356.17	6	1	6	77.52	1.813	−3.069	3.73
**M8**	372.17	7	1	7	86.75	2.073	−3.247	3.83
**M9**	386.18	7	1	8	86.75	2.631	−3.528	3.97
**M10**	328.18	5	1	5	60.45	2.587	−3.418	3.67
**M11**	314.16	5	1	5	60.45	1.857	−2.910	3.56
**M12**	385.20	7	2	8	89.55	1.394	−2.496	3.89

MW—molecular weight. Optional: 100–600; HBA—number of hydrogen bond acceptors. Optional: 0–12; HBD—number of hydrogen bond donors. Optional: 0–7; Nrot—number of rotatable bonds. Optional: 0–11; TPSA—topological polar surface area. Optional 0–140; logP—logarithm of the n-octanol/water distribution coefficients at pH = 7.4. Optional 0–3; logS—logarithm of aqueous solubility. Compounds with a high logS > −1 (as very good), −2 to −3 (moderate), and <−4 (as very poor).

**Table 9:** Absorption and distribution parameters for the designed compounds **M1**–**M12** and the reference structure **M0**, determined using the ADMETlab 3.0 program.

Property	Caco-2	MDCK	Pgp Inhibitor	Pgp Substrate	Plasma Protein-Binding Parameter
**M0**	−5.154	−4.794	---	+++	98.89%
**M1**	−5.030	−4.858	---	-	61.40%
**M2**	−4.958	−4.652	-	+	83.10%
**M3**	−4.987	−4.633	---	--	80.30%
**M4**	−4.925	−4.671	+	-	89.80%
**M5**	−5.117	−4.623	---	-	74.90%
**M6**	−5.117	−4.984	---	---	91.20%
**M7**	−4.987	−4.764	+	---	91.00%
**M8**	−5.008	−4.716	++	--	97.30%
**M9**	−4.990	−4.698	+	---	96.50%
**M10**	−4.934	−4.612	++	---	95.20%
**M11**	−4.902	−4.611	-	+	95.60%
**M12**	−4.923	−4.615	---	+++	82.50%

Caco-2—Caco-2 permeability. Optimal: higher than −5.15 log unit; MDCK—MDCK permeability. Low permeability: <2 × 10^−6^ [cm/s], medium permeability: 2–20 × 10^−6^ [cm/s] and high permeability: >20 × 10^−6^ [cm/s]; Pgp-inhibitor—P-glycoprotein inhibitor, classified with two groups: Category 1: Inhibitor (+++); Category 0: non-inhibitor (---), the output value is the probability of being Pgp-inhibitor (+++) within the range of --- to +++.; Pgp-substrate—P-glycoprotein substrate, classified with two groups: Category 1: substrate (+++), Category 0: non-substrate (---), the output value is the probability of being Pgp-substrate (+++), within the range of --- to +++.

## Data Availability

The original contributions presented in this study are included in the article/Supplementary Materials. Further inquiries can be directed to the corresponding author(s).

## Supplementary Materials

The following supporting information can be downloaded at: https://www.mdpi.com/article/10.3390/ijms26104620/s1. References [44,45,46,47,48,49,50,51,52] are cited in the Supplementary Materials.
