# Supplementary material for: In Silico Approach to Design of New Multi-Targeted Inhibitors Based on Quinoline Ring with Potential Anticancer Properties
Source: Int J Mol Sci. 2025 May 12;26(10):4620. doi: 10.3390/ijms26104620 (PMC12110963; doi:10.3390/ijms26104620)
Supplement: Supplementary file 1 [file ijms-26-04620-s001.zip › ijms-3608261-supplementary.pdf]

Supplementary Materials

The supplementary materials are available online. Supporting information includes characterization of molecular targets, ADMET properties of the compounds M0-M12 described in this article and molecular dynamics results.

Topoisomerase I

Topoisomerases are enzymes essential for the optimal functioning and proliferation of cells, including those involved in cancer. Topoisomerase I (TOPO-I) is comprised of three distinct domains. The N-terminus contains the DNA-binding region of the strand, while the C-terminus contains the catalytic region. Domains I, II, and III are implicated in the processes of DNA relaxation [44]. Topoisomerases regulate the packing of chromatin and facilitate the repair of DNA superstructures. The structure of the DNA double helix is susceptible to a variety of disorders. The unwinding of the double helix can result in the formation of underwinding or overwinding in adjacent regions, which in turn generates superhelical strain. If this situation is not resolved, it will impair the processes of replication, transcription, and chromosome segregation by inhibiting the operation of replication forks. The correct functioning of topoisomerase is crucial for the maintenance of genome integrity [45,46]. Nevertheless, the capacity to impede these enzymes or inflict damage represents a viable approach in cancer therapy. Topoisomerase inhibitors represent a significant component of chemotherapy regimens and form the foundation for numerous chemotherapy combinations employed in the treatment of a diverse range of cancers [47]. The structure is presented in Figure S1.

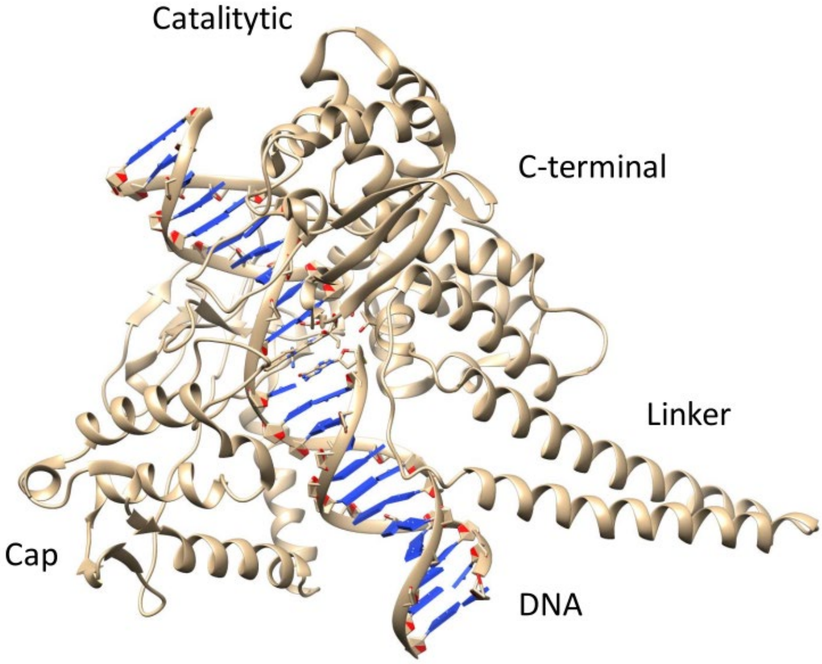

Figure S1. The structure of TOPO I.

Bromodomain 4

Proteins belonging to the bromodomain and extraterminal domain (BET) family are regarded as one of the most significant transcription regulators. The pharmacological inhibition of BET proteins is emerging as a promising therapeutic strategy for a range of diseases, including cancer and inflammatory disorders [48]. Of the proteins that comprise the BET family, Bromodomain 4 (BRD4) is the most well-studied. It plays a pivotal role as a transcription coactivator and functions as the primary elongation factor. It exists in three isoforms: BRD4L (the long isoform of bromodomain 4), BRD4Sa, and BRD4Sb (the short isoforms of bromodomain 4), which, based on the current state of knowledge, appear to perform distinct functions in the transcription process. Furthermore, it can function as a chromatin scaffold and participate in its reconstruction. Recent studies have indicated that BRD4 may promote the abnormal expression of oncogenes, including c-Myc, Aurora B, and Bcl-2. The presence of BRD4 is frequently indispensable for the expression of oncogenes that regulate cancer cells in hematological malignancies, multiple myeloma, acute myeloid, and lymphoblastic leukemia [49]. It has been proposed that the inhibition of BET proteins may induce the aging of cancer cells, which could result in increased recognition and phagocytosis by natural killer cells (NK) and macrophages. Inhibition of BET proteins has been demonstrated to markedly impair the proliferation of cancer cells, with a concomitant negative impact on the cell cycle and induction of apoptosis. These characteristics render BRD4 an appealing therapeutic target for anticancer therapy. In research, BRD4 inhibitors have been demonstrated to exhibit substantial antitumor activity in the treatment of triple-negative breast cancer, particularly by reducing cell invasion and migration, which limits metastasis, as well as by reducing IL-6-induced signaling through the so-called Jagged1/Notch1 pathway. Furthermore, they impede the progression of non-small cell lung cancer [49]. The structure is presented in Figure S2.

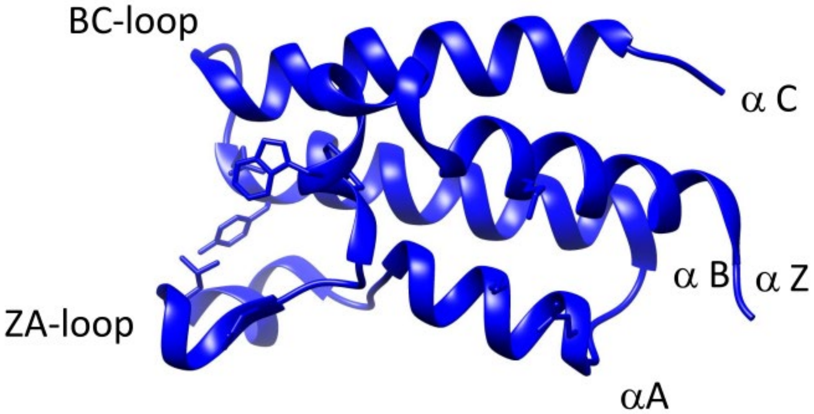

Figure S2. The structure of Bromodomain 4.

ATP-binding cassette sub-family G member 2 protein

The ATP-binding cassette sub-family G member 2 protein (ABCG2), also known as BCRP (breast cancer resistance protein), is a member of the ABC (ATP-binding cassette protein) family. It is a translocation protein that actively transports drugs and other compounds across the membrane against the concentration gradient, utilizing the energy derived from ATP hydrolysis [50,51]. It has been demonstrated that increased protein expression occurs in many cancers, particularly those that are highly resistant, as well as those that are particularly sensitive to topoisomerase inhibitors. The overexpression of this protein has been observed in malignant hematopoietic and lymphoid cells. It is noteworthy that the so-called side cell population, comprising cells that exhibit stem cell-like characteristics and express ABCG2, has been observed in various types of solid tumors, including those of the head, neck, breast, lung, ovary, pancreas, and colon. ABCG2 plays a significant role in developing resistance to chemotherapeutics, particularly mitoxantrone and camptothecin derivatives. The most recent reports indicate that the transporter is directly involved in the development of leukemia. Inhibition of the protein may result in elevated concentrations of anticancer drugs within cancer cells, thereby prolonging their efficacy. Furthermore, it may disrupt the normal functioning of the tumor and the accumulation of toxic substances within the cell [51–52]. As mentioned above, effective cancer treatment requires targeting multiple molecular pathways to overcome resistance and improve therapeutic efficacy. A promising strategy is the synergistic inhibition of TOPO-I, BRD4, and ABCG2, which involves the targeting of three critical mechanisms in cancer progression. The structure is presented in Figure S3.

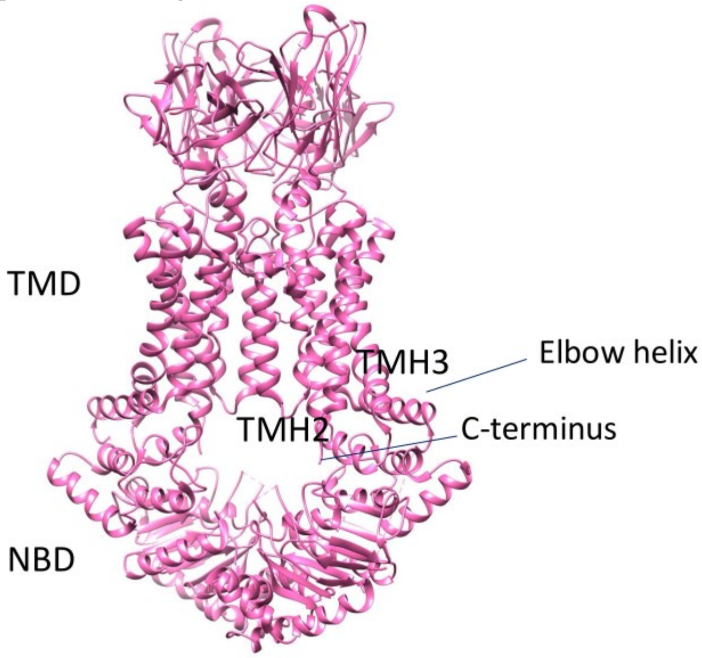

Figure S3. The structure of ABCG2.

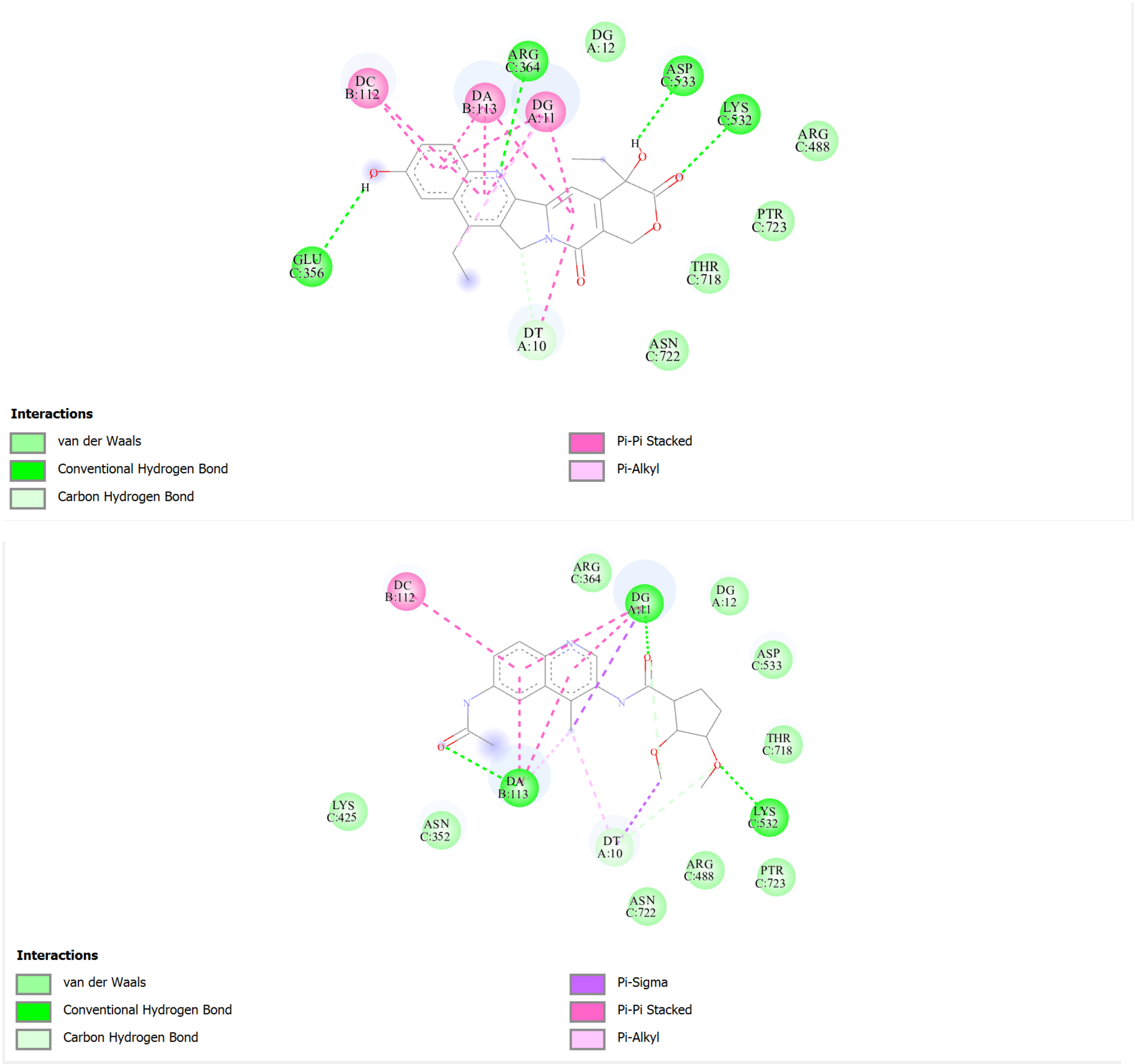

Figure S4. Intermolecular interactions of TOPO-I with M0 (top) and M3 (bottom).

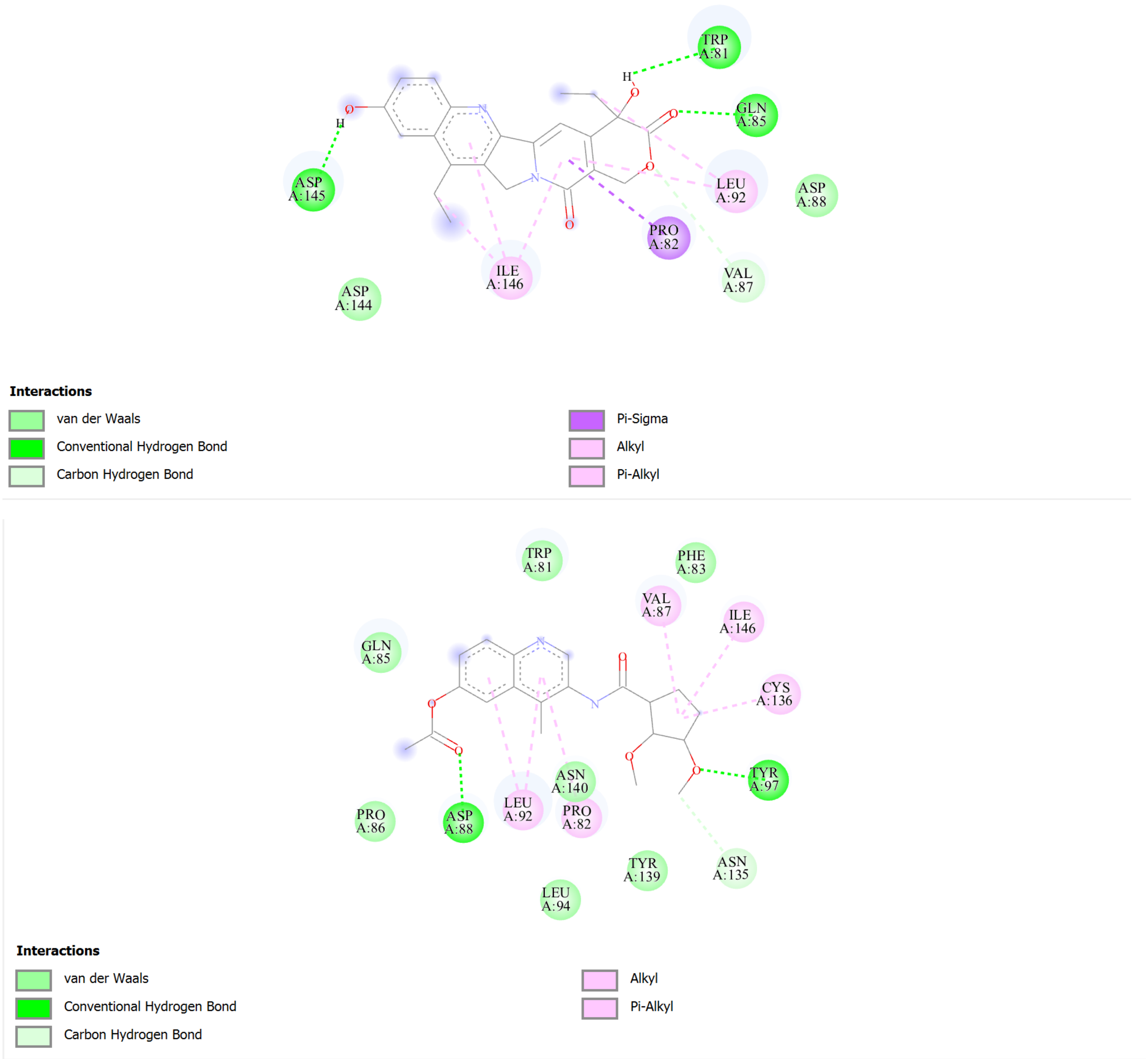

Figure S5. Intermolecular interactions of BRD4 with M0 (top) and M2 (bottom).

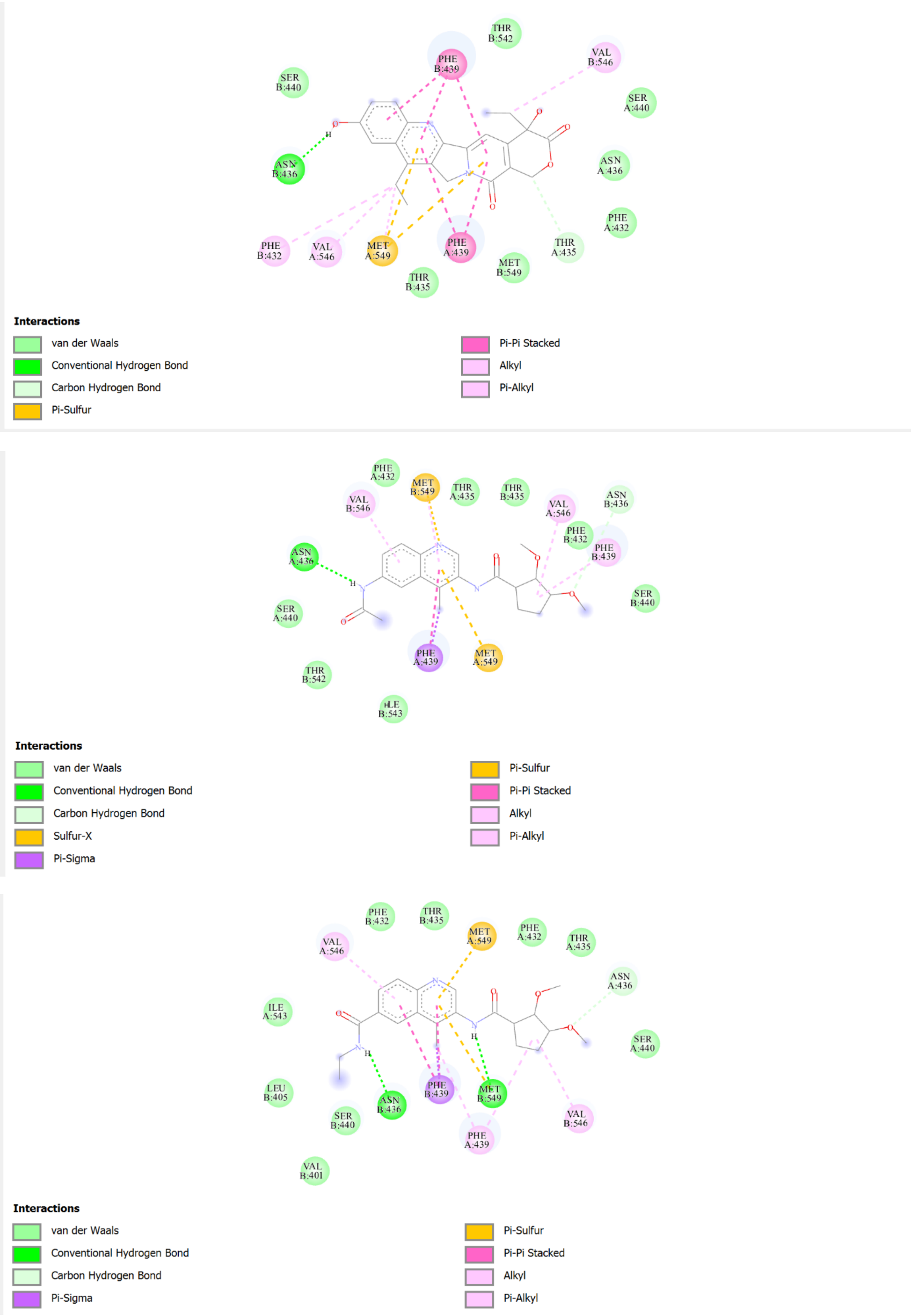

Figure S6. Intermolecular interactions of ABCG2 with M0 (top), M03 (center) and M12 (bottom).

Table S1. Distribution and excretion parameters for the designed compounds M0-M12, determined using ADMETLab 3.0 program.

Property	PPB	BBB Penetration	$T_{1/2}$ [h]
**M0**	98.89%	+	1.029
**M1**	61.40%	--	1.489
**M2**	83.10%	---	0.702
**M3**	80.30%	---	0.722
**M4**	89.80%	---	0.795
**M5**	74.90%	---	1.143
**M6**	91.20%	---	1.477
**M7**	91.00%	---	0.683
**M8**	97.30%	---	0.745
**M9**	96.50%	---	0.587
**M10**	95.20%	---	0.713
**M11**	95.60%	-	0.971
**M12**	82.50%	---	0.925

PPB – Plasma protein binding, **BBB** – blood-brain barrier, classified with two groups: Category 1: BBB (+++), Category 0: BBB(---), the output value is the probability of being BBB (+++), within the range of --- to +++; $\mathbf{T_{1/2}}$ – half life.

Table S2. Metabolism parameters for the designed compounds M0-M12, determined using the ADMETLab 3.0 program.

Property	CYP2C19		CYP2D6		CYP3A4	
	Inh.	Sub.	Inh.	Sub.	Inh.	Sub.
**M0**	---	-	-	-	++	---
**M1**	---	+++	---	-	--	++
**M2**	---	--	---	---	---	--
**M3**	---	+++	---	---	++	+++
**M4**	-	+++	---	-	++	++
**M5**	---	---	---	---	---	---
**M6**	---	---	---	---	---	---
**M7**	-	-	---	--	-	-
**M8**	---	---	---	---	---	---
**M9**	++	---	---	---	--	++
**M10**	-	+++	---	++	++	+++
**M11**	+	+++	---	--	--	+++
**M12**	---	++	---	---	---	---

Inh. – inhibitor, classified with two groups: Category 1: Inhibitor (+++), Category 0: Non-inhibitor (---). The output value is the probability of being inhibitor (+++), within the range of --- to +++; **Sub** – substrate, classified with two groups: Category 1: Substrate(+++), Category 0: Non-substrate(---). The output value is the probability of being substrate (+++), within the range of --- to +++.

Table S3. Toxicity parameters for the designed compounds M0-M12, determined using ADMETLab 3.0 program.

Property	Carcinogenicity	hERG blockers	Respiratory toxicity	Neurotoxicity	Ototoxicity	H-HT
**M0**	0.991	0.109	0.455	0.985	0.773	0.959
**M1**	0.531	0.144	0.699	0.112	0.655	0.599
**M2**	0.478	0.211	0.462	0.519	0.413	0.536
**M3**	0.700	0.221	0.260	0.443	0.326	0.850
**M4**	0.730	0.541	0.718	0.699	0.586	0.537
**M5**	0.784	0.449	0.392	0.794	0.464	0.686
**M6**	0.502	0.177	0.419	0.432	0.720	0.690
**M7**	0.713	0.331	0.483	0.581	0.575	0.744
**M8**	0.719	0.399	0.388	0.593	0.540	0.182
**M9**	0.724	0.414	0.121	0.293	0.460	0.077
**M10**	0.620	0.298	0.451	0.576	0.481	0.654
**M11**	0.602	0.382	0.660	0.615	0.486	0.471
**M12**	0.691	0.588	0.396	0.651	0.573	0.497

Carcinogenicity – classified with two groups: Category 1: carcinogens; Category 0: non-carcinogens. The output value is the probability of being toxic. **hERG blockers** – molecules with IC50 ≤ 10μM or ≥50% inhibition at 10μM classified as hERG+ (Category 1). The output value is the probability of being hERG+, within the range of 0 to 1. **Respiratory toxicity** – classified with two groups: Category 1: respiratory toxicants; Category 0: non-respiratory toxicants. The output value is the probability of being toxic within the range of 0 to 1. **Neurotoxicity** – drug-induced neurotoxicity classified with two groups: Category 0: non-neurotoxic; Category 1: neurotoxic. The output value is the probability of being neurotoxic, within the range of 0 to 1. **Ototoxicity** – classified with two groups: Category 0: non-ototoxicity; Category 1: ototoxicity. The output value is the probability of being ototoxicity, within the range of 0 to 1. **H-HT** – human hepatotoxicity classified with two groups: Category 0: H-HT negative; Category 1: H-HT positive. The output value is the probability of being toxic, within the range of 0 to 1.

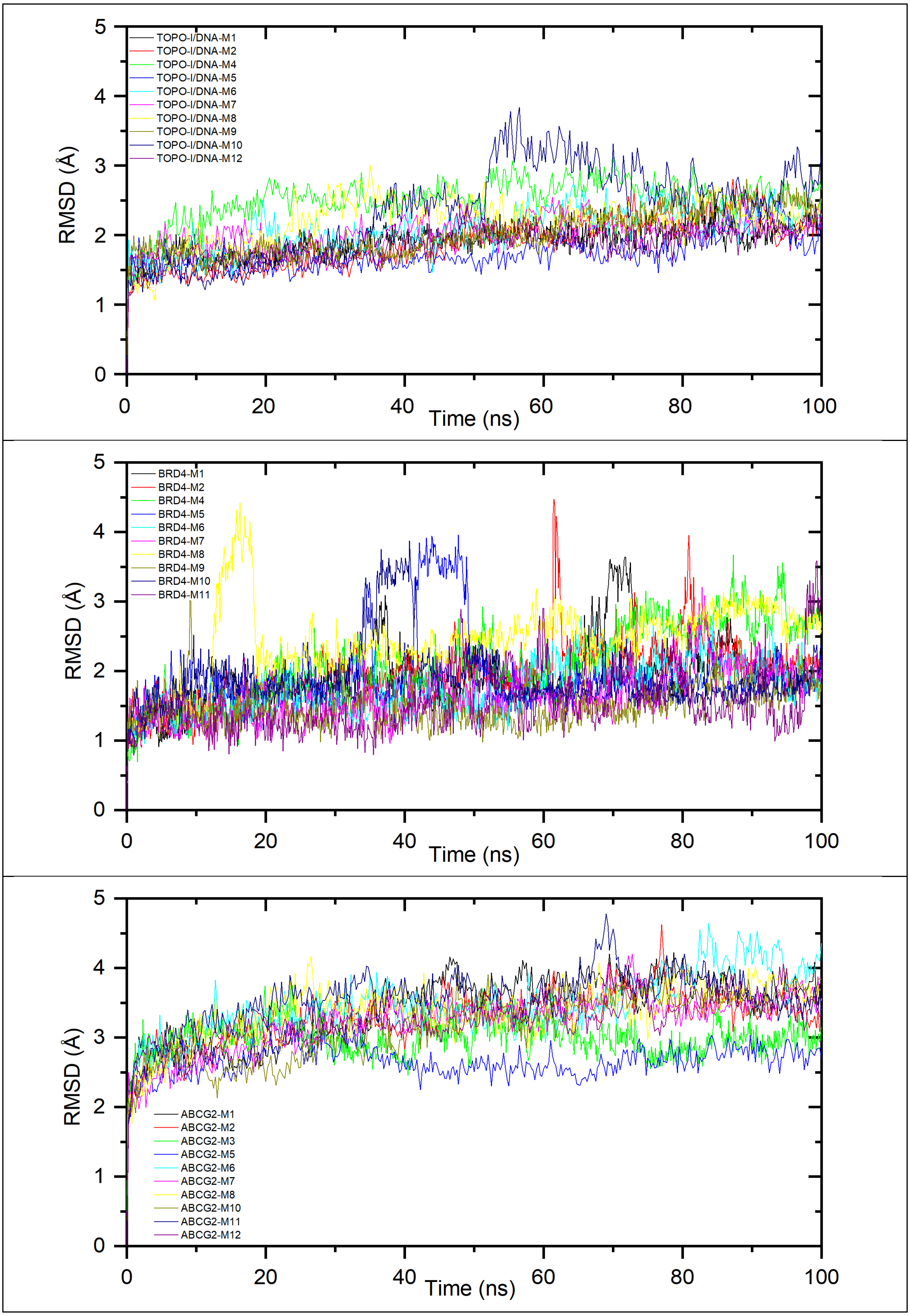

Figure S7. The RMSD plot of the protein backbone after least squares fitting to the protein backbone for complexes with TOPO-I/DNA, BRD4 and ABCG2.
